# Plasma chondroitin sulfate predicts the effectiveness of fluid resuscitation strategies in patients with sepsis

**DOI:** 10.1172/JCI202480

**Published:** 2026-02-03

**Authors:** Kaori Oshima, Bailu Yan, Ran Tao, Gustavo Amorim, Chiara Di Gravio, Sarah A. McMurtry, Ryan C. Burke, Yunbi Nam, Ina Nikolli, Max S. Kravitz, Daniel Stephenson, Aaron Issaian, Kirk C. Hansen, Angelo D’Alessandro, Ivor S. Douglas, Wesley H. Self, Christopher J. Lindsell, Carolyn Leroux, Angelika Ringor, Michael A. Matthay, Jonathan S. Schildcrout, Nathan I. Shapiro, Eric P. Schmidt

**Affiliations:** 1 Department of Medicine, Mass General Brigham, Boston, Massachusetts, USA.; 2 Department of Biostatistics and; 3 Vanderbilt Genetics Institute, Vanderbilt University Medical Center, Nashville, Tennessee, USA.; 4 Department of Epidemiology and Biostatistics, School of Public Health, Imperial College London, London, United Kingdom.; 5 Department of Medicine, University of Colorado, Aurora, Colorado, USA.; 6 Department of Emergency Medicine, Beth Israel Deaconess Medical Center and Harvard Medical School, Boston, Massachusetts, USA.; 7 Department of Biochemistry and Molecular Genetics, University of Colorado, Aurora, Colorado, USA.; 8 Department of Medicine, Denver Health Medical Center, Denver, Colorado, USA.; 9 Vanderbilt Institute for Clinical and Translational Research and Department of Emergency Medicine, Vanderbilt University Medical Center, Nashville, Tennessee, USA.; 10 Department of Biostatistics and Bioinformatics and Duke Clinical Research Institute, Duke University Medical Center, Durham, North Carolina, USA.; 11 Department of Medicine, Cardiovascular Research Institute, University of California, San Francisco, San Francisco, California, USA.

**Keywords:** Clinical Research, Infectious disease, Inflammation, Biomarkers, Glycobiology

## Abstract

**BACKGROUND:**

Plasma heparan sulfate, a glycosaminoglycan released during endothelial glycocalyx degradation, predicts sepsis mortality. Chondroitin sulfate is a circulating glycosaminoglycan not specific to glycocalyx degradation; its relevance to sepsis is unknown.

**METHODS:**

We studied the associations of plasma chondroitin sulfate with (a) mortality in patients with sepsis-associated hypotension and (b) the relative effectiveness of a randomly assigned liberal versus restrictive intravenous fluid resuscitation strategy. We selected 574 patients enrolled in the Crystalloid Liberal or Vasopressors Early Resuscitation in Sepsis trial using an outcome-enriched sampling strategy. We used liquid chromatography–mass spectrometry to quantify plasma chondroitin sulfate. In comparison, we measured hyaluronic acid as a glycocalyx degradation marker and IL-6 as an inflammatory biomarker. We conducted Cox proportional hazards regression analyses to examine associations of baseline biomarker concentrations with mortality and resuscitation strategy effectiveness. We used inverse probability of selection weights and generalized raking to account for the nonrepresentative sampling design.

**RESULTS:**

Plasma chondroitin sulfate, hyaluronic acid, and IL-6 were associated with mortality within 90 days. As baseline chondroitin sulfate increased, subsequent randomization to a restrictive strategy was increasingly beneficial (*P* = 0.022): treatment effect hazard ratio (restrictive versus liberal) for mortality was estimated as 1.49 (95% CI, 0.98–2.27), 1.30 (95% CI, 1.00–1.69), 1.09 (95% CI, 0.82–1.44), 0.88 (95% CI, 0.66–1.16), and 0.71 (95% CI, 0.52–0.97) for 10th, 25th, 50th, 75th, and 90th percentiles of baseline chondroitin sulfate.

**CONCLUSION:**

Plasma chondroitin sulfate predicts sepsis mortality and may modify the response to a subsequent liberal versus restrictive intravenous fluid resuscitation strategy.

**TRIAL REGISTRATION:**

ClinicalTrials.gov NCT03434028.

**FUNDING:**

NIH grants R01HL149422 and R01HL094786.

## Abstract 2

In patients with sepsis and hypotension, plasma concentrations of chondroitin sulfate predicted the subsequent response to a randomized liberal or restrictive intravenous fluid resuscitation strategy.

## Introduction

Randomized controlled trials comparing liberal and restrictive intravenous fluid resuscitation strategies in sepsis have found that neither strategy confers mortality benefit over the other in an unselected patient population (1, 2). However, it remains unclear if these strategies are equally effective for all patients or if benefits experienced by a specific patient subtype for a given fluid resuscitation strategy are offset by harms experienced by a different patient subtype for that same strategy. Increasing appreciation of the biological heterogeneity of sepsis suggests that the ideal intravenous fluid resuscitation strategy for a patient with sepsis may vary based on the specific pathobiology responsible for the development of critical illness in that patient (3). To achieve such a precision medicine approach to fluid resuscitation, there is a need to identify predictive biomarkers that can identify an individual’s responsiveness to a subsequent resuscitation strategy. To date, no such biomarker has been identified.

Circulating glycosaminoglycans, such as heparan sulfate and hyaluronic acid, serve as blood biomarkers of pathological shedding of endothelial glycocalyx, a glycosaminoglycan-enriched endovascular layer necessary for vascular homeostasis (4). Accordingly, elevated plasma glycocalyx degradation products have been independently associated with worsened outcomes in patients with sepsis (5–7). Chondroitin sulfate, the most abundant glycosaminoglycan in human plasma (8), is similarly a glycocalyx glycosaminoglycan that is shed during endothelial glycocalyx degradation (9–11). However, the majority of plasma chondroitin sulfate is not derived from the glycocalyx but rather exists as components of circulating proteoglycans, such as bikunin, a constituent of the circulating antiprotease inter-α-trypsin inhibitor (12, 13). Reflecting these multifaceted origins, the value of plasma chondroitin sulfate as a prognostic and/or predictive biomarker in sepsis is uncertain.

To study the prognostic or predictive abilities of chondroitin sulfate as a biomarker in sepsis, we leveraged plasma specimens and patient data collected as part of a prespecified ancillary study of the Crystalloid Liberal or Vasopressors Early Resuscitation in Sepsis (CLOVERS) trial, which randomized 1563 patients with sepsis-induced hypotension to a liberal intravenous fluid resuscitation strategy or a restrictive fluid strategy accompanied by early vasopressor administration (ClinicalTrials.gov NCT03434028) (2). We sought to determine if plasma chondroitin sulfate concentrations were associated with (a) mortality within 90 days of study randomization and (b) differential treatment effectiveness of the liberal and restrictive fluid strategies. We compared these prognostic and predictive abilities of chondroitin sulfate to hyaluronic acid, a plasma glycosaminoglycan studied as a specific biomarker of endothelial glycocalyx degradation (9, 14), and IL-6, a marker of systemic inflammation (15).

## Results

### Study cohorts.

CLOVERS inclusion criteria included patients aged >18 years with sepsis-induced hypotension, defined as systolic blood pressure <100 mmHg after 1 liter of intravenous fluid, who were early in their hospital course. The complete inclusion and exclusion criteria for the trial are available in the primary CLOVERS report (2). Plasma specimens were collected at enrollment (a median of 61 minutes after study eligibility) and 24 and 72 hours after randomization.

Since liquid chromatography–mass spectrometry quantification of chondroitin sulfate and hyaluronic acid is cost-prohibitive to perform on all CLOVERS participants, we selected a subset of 600 participants from the 1,563 participants for inclusion into the present substudy. As previously described (7), we enriched this subset with patients who died within 90 days of randomization and those who had acute respiratory distress syndrome (ARDS) at baseline or within 7 days of randomization. By enriching the subset with those who died, we observed a relatively informative sample compared with a random sample and therefore can estimate the parameters in our models with lower uncertainty (i.e., smaller standard errors) and higher power. We then randomly selected from the remaining participants to reach a total of 300 participants from each of the liberal and restrictive groups. Twenty participants did not have sufficient baseline plasma sample volume available for mass spectrometry, and 6 participants were missing baseline characteristics. Our analyses were therefore performed using samples collected from the remaining 574 participants. In those who were still alive and hospitalized, repeat measurements were made 24 hours (*N* = 476) and 72 hours (*N* = 326) after study randomization. Since the selected subsamples were not random samples from the original CLOVERS cohort, the analyses were weighted using inverse probability of selection weighting and generalized raking, so that results from our selected cohort generalize to the population represented by the entire CLOVERS cohort (Table 1 and Supplemental Table 1; supplemental material available online with this article; https://doi.org/10.1172/JCI202480DS1).

IL-6 measurements were performed as part of the trial protocol on 1,371 of the 1,563 participants enrolled in CLOVERS. There were 2 participants missing baseline characteristics, so our analyses were performed on the remaining 1,369 participants. IL-6 was measured at enrollment (*N* = 1,369) and 72 hours (*N* = 844). Like the analyses of chondroitin sulfate and hyaluronic acid, we used inverse probability of selection weighting and generalized raking estimation strategies to ensure that results from IL-6 analyses generalize to the population represented by the full CLOVERS cohort (Supplemental Table 2).

### Association of chondroitin sulfate with indices of endothelial glycocalyx degradation and systemic inflammation.

**Figure 1:**
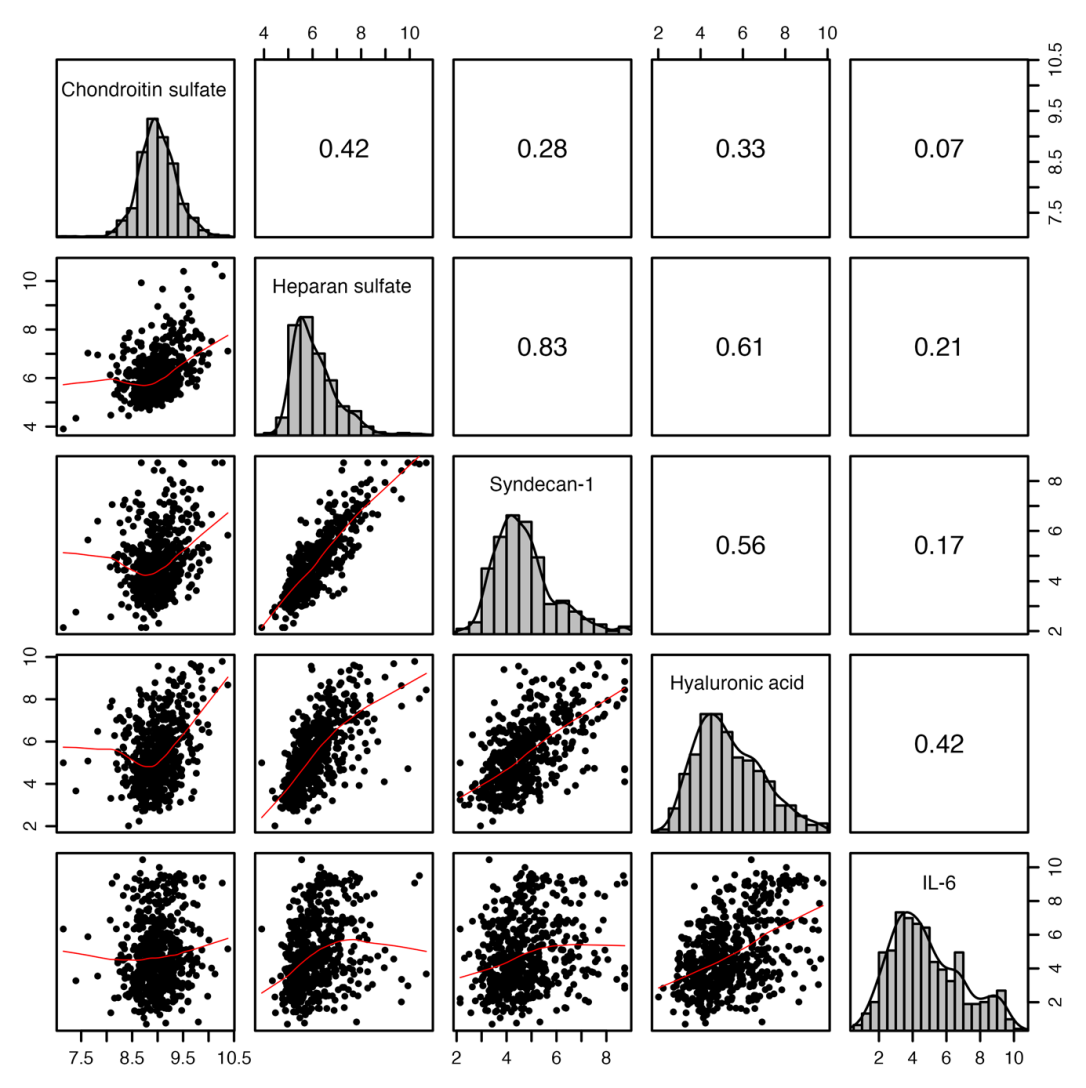
Correlation among chondroitin sulfate, indices of endothelial glycocalyx degradation (hyaluronic acid, heparan sulfate, and syndecan-1), and an index of systemic inflammation (IL-6) in plasma samples collected at CLOVERS enrollment. We display scatterplots of the observed joint distributions of the log-transformed biomarker concentrations at baseline in the lower triangular paired panels. The histogram and density curves of the log of biomarker concentrations are shown on the diagonals. Weighted Pearson correlation coefficients are shown in the corresponding upper triangular paired panels.

In each plasma sample collected for mass spectrometry analysis of circulating glycosaminoglycans, we compared concentrations of chondroitin sulfate, hyaluronic acid, and IL-6 with previously measured concentrations of heparan sulfate and syndecan-1 (7), canonical markers of endothelial glycocalyx shedding that we have previously shown to be prognostic for 90-day mortality in CLOVERS (7). We found moderate associations between circulating chondroitin sulfate and canonical glycocalyx shedding markers hyaluronic acid (*r* = 0.33) and heparan sulfate (*r* = 0.42) and the glycocalyx proteoglycan syndecan-1 (*r* = 0.28) in samples collected at study enrollment, confirming that circulating chondroitin sulfate is not solely a marker of endothelial glycocalyx degradation (Figure 1). Chondroitin sulfate was not well correlated with IL-6 (*r* = 0.07), a canonical marker of systemic inflammation in sepsis (Figure 1). Taken together, these findings indicate that plasma chondroitin sulfate reflects biological processes distinct from endothelial glycocalyx injury and/or systemic inflammation.

### Plasma chondroitin sulfate, hyaluronic acid, and IL-6 at study enrollment are independently associated with sepsis mortality.

**Figure 2:**
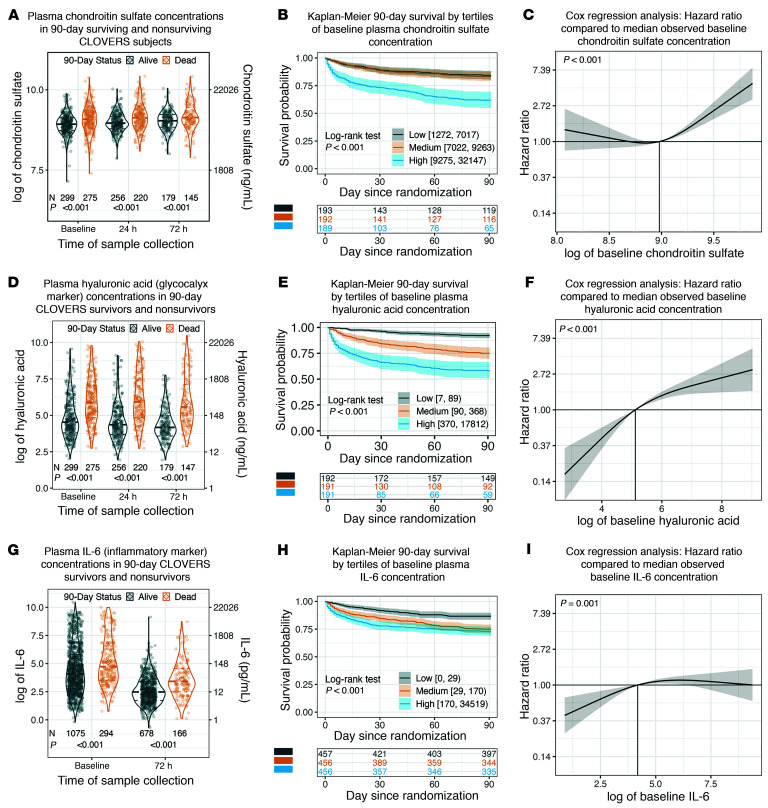
Plasma biomarkers as predictors of 90-day mortality in CLOVERS. (**A**) Plasma chondroitin sulfate, (**D**) hyaluronic acid, and (**G**) IL-6 (measured only at 72 hours) were elevated at all time points in patients who died within 90 days of CLOVERS enrollment. The number of observations at each time point and *P* values from the Wald test of inverse probability weighted linear regression are shown under the violin plots. (**B**) Kaplan-Meier plot of the unadjusted association between baseline plasma chondroitin sulfate, divided into tertiles, and survival. Tertile ranges are listed in the legend, with the table beneath the plot indicating the number of observed participants at risk at each time. Kaplan-Meier estimates and the log rank test were calculated using inverse probability of selection weights. (**C**) Covariate-adjusted association of baseline (log-transformed) chondroitin sulfate with mortality rates within 90 days of randomization. To address concerns about violations of the proportional hazards assumption, this Cox model and all other Cox models are stratified by sex assigned at birth and chronic heart failure. Additionally, we estimated associations between SOFA score and log-hazard of death separately for 0–4 days, 5–11 days, and 12–90 days of follow-up. Due to the stratified sampling study design, we used generalized raking when fitting the model. Identical analyses were conducted to estimate the association between baseline plasma hyaluronic acid (**E** and **F**), IL-6 (**H** and **I**), and mortality rates within 90 days of randomization. Full covariate-adjusted models are demonstrated in Supplemental Figures 1–3.

At each time point measured, plasma concentrations of chondroitin sulfate (Figure 2A) were elevated in CLOVERS patients who died within 90 days of study randomization. Patients in the highest tertile of plasma chondroitin sulfate concentration (9,275–32,147 ng/mL) at the time of study enrollment (i.e., prior to randomization) had a significantly higher 90-day mortality compared with those in the medium (7,022–9,263 ng/mL) or low (1,272–7,017 ng/mL) plasma chondroitin sulfate tertiles (log-rank *P* < 0.001) (Figure 2B). The association of elevated plasma chondroitin sulfate at study enrollment with increased 90-day mortality persisted in our fitted Cox model that adjusted for severity of illness, patient demographics, randomized fluid resuscitation strategy, and chronic comorbidities (Figure 2C, entire model shown in Supplemental Figure 1). For example, the hazard ratio for mortality comparing the 10th percentile (5,303 ng/mL) to the 50th percentile (7,861 ng/mL) of baseline chondroitin sulfate was 1.06 (95% CI, 0.84–1.33), while the hazard ratio comparing the 90th percentile (12,202 ng/mL) to the 50th percentile was 1.98 (95% CI, 1.65–2.39).

Similar to chondroitin sulfate, plasma concentrations of hyaluronic acid (a canonical endothelial glycocalyx degradation marker) and IL-6 (a canonical marker of systemic inflammation) were associated with 90-day survival. Patients who went on to die within 90 days of study enrollment had higher levels of plasma hyaluronic acid at all time points (Figure 2D) in comparison to 90-day survivors. Patients in the highest tertile of plasma hyaluronic acid concentration (370–17,812 ng/mL) at the time of study enrollment demonstrated higher 90-day mortality than those in the medium (90–368 ng/mL) and low (7–89 ng/mL) tertiles (log-rank *P* < 0.001) (Figure 2E). Our adjusted Cox model accordingly showed that baseline hyaluronic acid was independently associated with 90-day mortality (Figure 2F, entire model shown in Supplemental Figure 2). The hazard ratio comparing the 10th percentile (31 ng/mL) to the 50th percentile (142 ng/mL) of baseline hyaluronic acid was 0.28 (95% CI, 0.17–0.47), while the hazard ratio comparing the 90th percentile (1321 ng/mL) to the 50th percentile was 2.05 (95% CI, 1.58–2.66). Similarly, patients who went on to die within 90 days of study enrollment had higher levels of plasma IL-6 at all time points (Figure 2G) in comparison to 90-day survivors. Patients in the lowest tertile of baseline plasma IL-6 (0–29 pg/mL) at study enrollment had lower mortality than those in the highest tertile (170–34,519 pg/mL) or medium tertile (29–170 pg/mL) tertiles (log-rank *P* < 0.001; Figure 2H). This association of baseline IL-6 with 90-day mortality persisted in covariate-adjusted analysis (Figure 2I, entire model shown in Supplemental Figure 3). The hazard ratio comparing the 10th percentile (8 pg/mL) to the 50th percentile (63 pg/mL) of baseline IL-6 was 0.60 (95% CI, 0.44–0.81), while the hazard ratio comparing the 90th percentile (2,382 pg/mL) to the 50th percentile was 1.08 (95% CI, 0.83–1.41).

### Postresuscitation plasma chondroitin sulfate, hyaluronic acid, and IL-6 are not altered by antecedent randomization to a liberal versus restrictive fluid resuscitation strategy.

**Figure 3:**
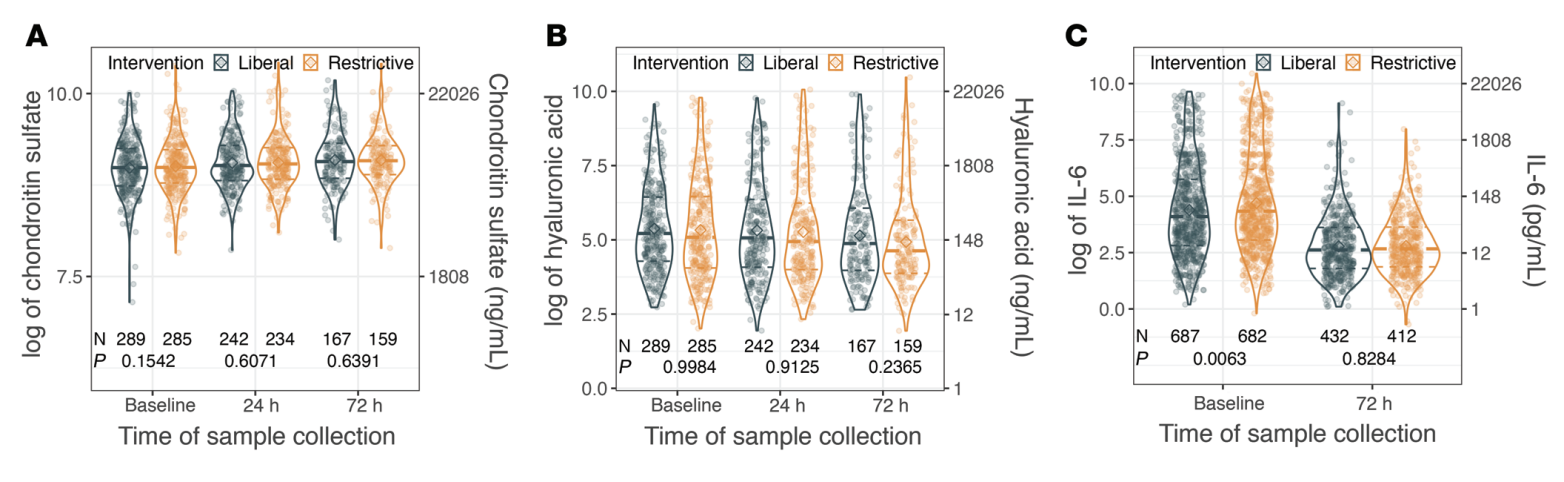
Plasma biomarker concentrations in CLOVERS patients randomized (after day 0 sample collection) to 24 hours of protocolized liberal or restrictive fluid resuscitation. On unadjusted analyses, treatment approach had no effect on subsequent (**A**) chondroitin sulfate, (**B**) hyaluronic acid, or (**C**) IL-6 (measured only at 72 hours) plasma concentrations. The number of observations at each time point and *P* values from the Wald test of inverse probability weighted linear regression are shown under the violin plots.

Previous observational studies suggested that a liberal intravenous fluid resuscitation strategy may induce iatrogenic endothelial glycocalyx degradation, leading to an increase in circulating glycocalyx fragments (16). However, such iatrogenic endothelial glycocalyx injury was not observed in randomized, prospective trials of fluid resuscitation (7). In contrast to glycocalyx-derived glycosaminoglycans, the effect of antecedent intravenous fluid resuscitation on circulating chondroitin sulfate concentrations was unstudied. We found that plasma chondroitin sulfate concentrations 24 hours after enrollment were unaffected by antecedent randomized resuscitation strategy (Figure 3A and Supplemental Figure 4), suggesting that choice of fluid resuscitation strategy did not induce changes in circulating chondroitin sulfate. Similarly, plasma hyaluronic acid (a canonical endothelial glycocalyx degradation biomarker) 24 hours after enrollment (i.e., at the completion of the study fluid protocol) was not affected by the antecedent randomization to either fluid resuscitation strategy (Figure 3B and Supplemental Figure 5), supporting previous studies’ findings that fluid resuscitation strategy does not drive glycocalyx injury.

As IL-6 levels were not immediately measured after completion of the 24-hour period of protocolized fluid resuscitation, we used plasma IL-6 measured 72 hours after enrollment to determine if antecedent early fluid resuscitation strategy influenced persistent systemic inflammation. Using our adjusted linear regression model, we found that early assignment to a liberal or restrictive intravenous fluid resuscitation strategy had no association with plasma IL-6 72 hours after study enrollment, suggesting that the early (first 24 hours) resuscitation strategy had no effect on persistent indices of systemic inflammation (Figure 3C and Supplemental Figure 6).

### Plasma chondroitin sulfate, but not hyaluronic acid or IL-6, modified the effectiveness of a subsequent, randomly assigned fluid resuscitation strategy on 90-day mortality.

**Figure 4:**
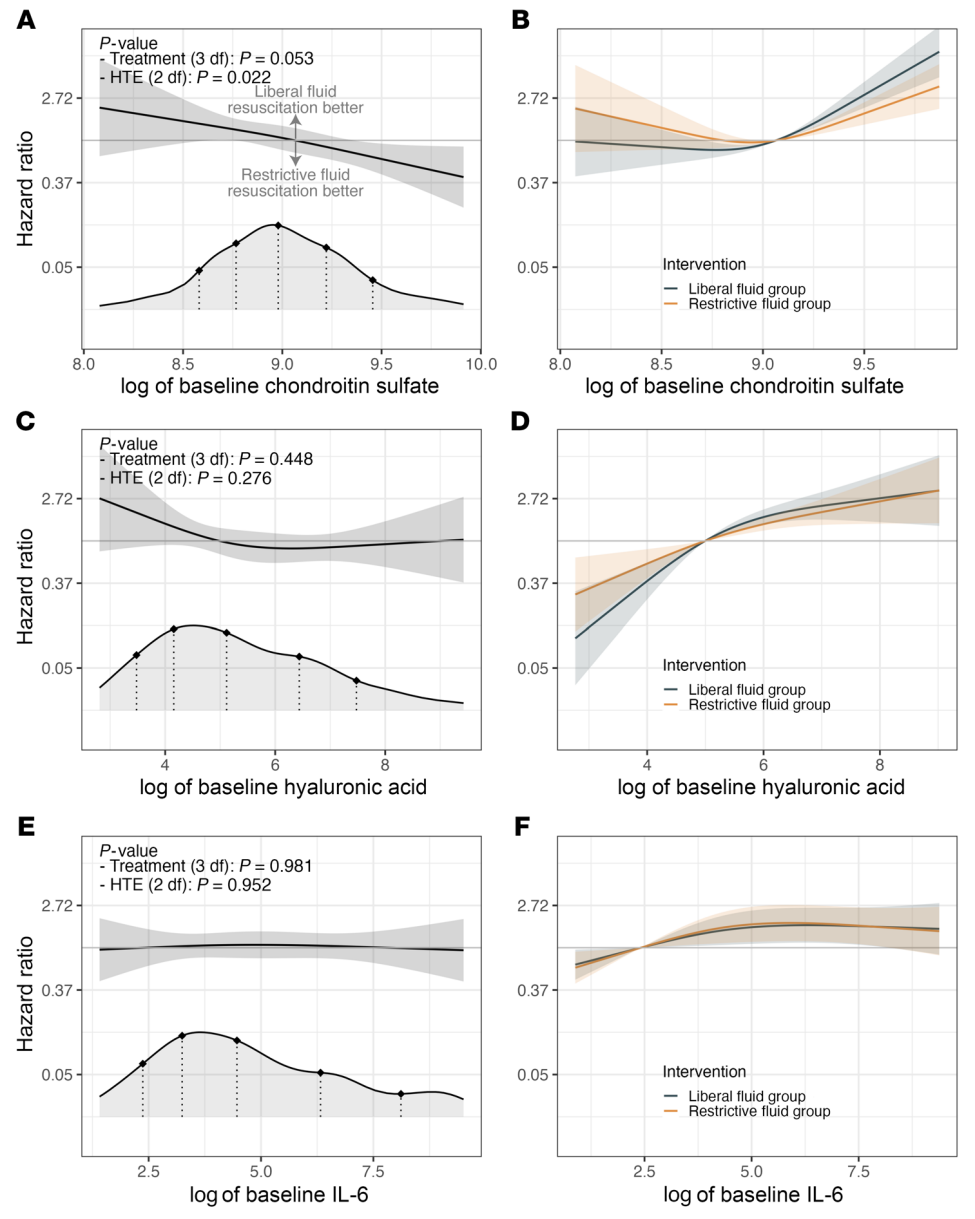
The effectiveness of the randomized treatment assignment is modified by (log of) baseline chondroitin sulfate but not hyaluronic acid or IL-6 plasma concentrations. Panels display results from models that added an interaction between randomized treatment assignment and baseline log-transformed plasma chondroitin sulfate (**A** and **B**), hyaluronic acid (**C** and **D**), or IL-6 (**E** and **F**) to the models shown in Figure 2, C, F, and I. Panels on the left demonstrate hazard ratios comparing restrictive versus liberal fluid resuscitation and pointwise 95% CIs across the distribution of log of baseline chondroitin sulfate (**A**), hyaluronic acid (**C**), or IL-6 (**E**) concentrations. The densities at the bottom of the panels refer to the distribution of baseline (log-transformed) plasma biomarker concentrations across the CLOVERS cohort, and for reference, we highlight the 10th, 25th, 50th, 75th, and 90th percentiles with dotted lines. We show the *P* values associated with (a) the overall treatment effect and (b) the heterogeneity of treatment effect. The heterogeneity of treatment effect test is a 2 degrees of freedom test Against the null hypothesis that the treatment effect is constant across the distribution of the biomarker values. The overall treatment effect test includes the main effect of the intervention (randomized fluid resuscitation strategy) and the 2 terms for the interaction with biomarker values. It is a test against the null hypothesis that treatment has no effect on mortality rates within 90 days of randomization against the alternative that it has any effect. Panels on the right demonstrate estimated hazard ratio and pointwise 95% CI comparing baseline log chondroitin sulfate (**B**), hyaluronic acid (**D**), or IL-6 (**F**) values to a reference value when the hazard ratio of the restrictive versus liberal fluid resuscitation strategies is equal to one. The associations are displayed separately for those randomized to the restrictive and liberal fluid resuscitation strategies.

We sought to determine whether baseline plasma chondroitin sulfate concentration modified the relative effectiveness of a subsequent, randomly assigned liberal versus restrictive intravenous fluid resuscitation strategy on mortality within 90 days. Our covariate-adjusted Cox model demonstrated a differential treatment effect (Figure 4A; *P* = 0.022) according to chondroitin sulfate concentrations at study enrollment. As baseline plasma chondroitin sulfate concentrations increased, randomization to a restrictive (as compared with liberal) intravenous fluid resuscitation strategy was estimated to be increasingly beneficial to patients. For example, the treatment effect hazard ratio (restrictive versus liberal fluid resuscitation strategy) for mortality was estimated to be 1.49 (95% CI, 0.98–2.27), 1.30 (95% CI, 1.00–1.69), 1.09 (95% CI, 0.82–1.44), 0.88 (95% CI, 0.66–1.16), and 0.71 (95% CI, 0.52–0.97) for baseline chondroitin sulfate concentrations of 5,329 ng/mL, 6,418 ng/mL, 7,937 ng/mL, 10,112 ng/mL, and 12,779 ng/mL, respectively. These reference values of baseline chondroitin sulfate concentrations represent the 10th, 25th, 50th, 75th, and 90th percentiles of the observed distribution. Associations of chondroitin sulfate concentrations with the hazard ratios for mortality are shown separately for the liberal and restrictive resuscitation strategy arms in Figure 4B.

In contrast to chondroitin sulfate, we observed no evidence of differential treatment effect for 90-day mortality as a function of baseline plasma hyaluronic acid (Figure 4, C and D) or IL-6 (Figure 4, E and F) and subsequent randomization to liberal or restrictive fluid resuscitation strategy.

### Disaccharide analyses suggest the importance of unsulfated chondroitin sulfate in modifying response to fluid resuscitation strategy.

**Figure 5:**
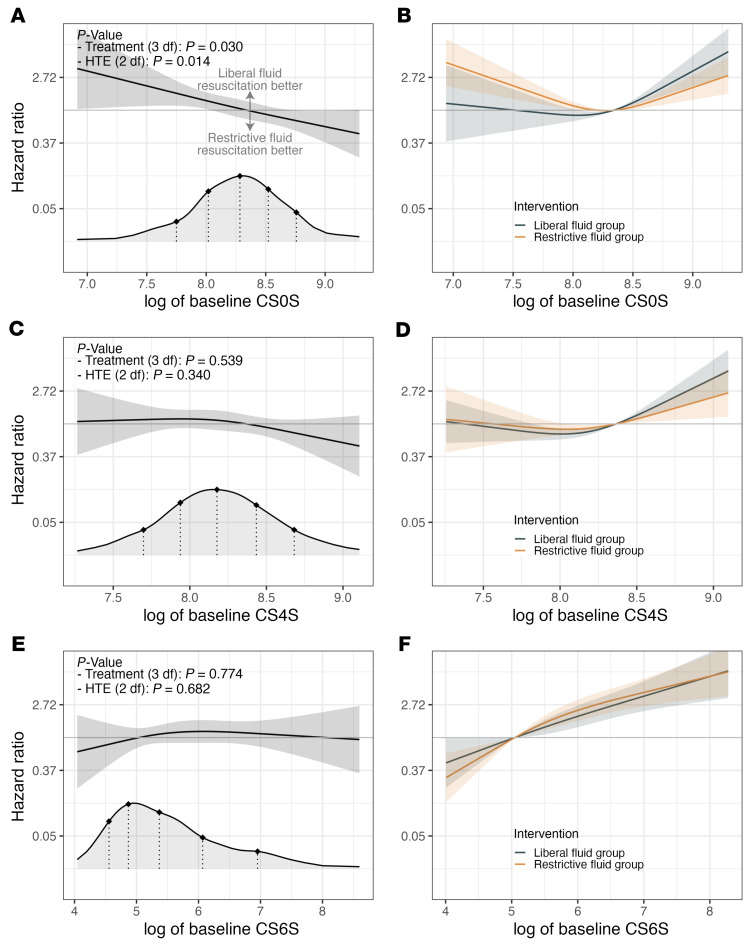
The effectiveness of the randomized treatment assignment is modified by (log of) baseline unsulfated chondroitin sulfate disaccharides but not 4-O or 6-O sulfated disaccharides. Panels on the left demonstrate hazard ratios comparing restrictive versus liberal fluid resuscitation and pointwise 95% CI across the distribution of log of baseline unsulfated (CS0S, **A**), 4-O sulfated (CS4S, **C**), or 6-O sulfated (CS6S, **E**) chondroitin sulfate concentrations, as per modeling detailed in Figure 4. The densities at the bottom of the panels refer to the distribution of baseline (log-transformed) plasma biomarker concentrations across the CLOVERS cohort, and for reference, we highlighted the 10th, 25th, 50th, 75th, and 90th percentiles with dotted lines. We show the *P* values associated with (a) the overall treatment effect and (b) the heterogeneity of treatment effect. Panels on the right demonstrate estimated hazard ratio and pointwise 95% CI comparing baseline log unsulfated (**B**), 4-O sulfated (**D**), or 6-O sulfated (**F**) chondroitin sulfate values to a reference value when the hazard ratio of the restrictive versus liberal fluid resuscitation strategies is equal to one.

To explore potential mechanisms underlying the predictive ability of circulating chondroitin sulfate on an individual patient’s response to a subsequent, randomly assigned fluid resuscitation strategy, we investigated the sulfation characteristics of plasma chondroitin sulfate in CLOVERS participants. Chondroitin sulfate is a linear polysaccharide, composed of repeating glucuronic acid-galactosamine disaccharides. Each disaccharide unit may be unsulfated (0S) or sulfated at either the 6-O or 4-O position of galactosamine. The relative abundance of these sulfated disaccharides within plasma chondroitin sulfate, as measured by mass spectrometry, can provide insight into the potential binding partners of circulating chondroitin sulfate during sepsis. For example, the circulating proteoglycan bikunin, a component of the endogenous antiprotease inter-α-trypsin inhibitor, is decorated with chondroitin sulfate that is enriched in 4-O sulfated disaccharides (12, 13). Interestingly, we found that the differential treatment effect of baseline plasma chondroitin sulfate on the response to a fluid resuscitation strategy was largely driven by unsulfated chondroitin sulfate (Figure 5, A and B) and not by 4-O (Figure 5, C and D) or 6-O (Figure 5, E and F) sulfated disaccharides. Therefore, the lack of predictive ability of 4-O chondroitin sulfate suggests that the observed differential response of fluid resuscitation strategy in CLOVERS might not be mediated by circulating bikunin. Patient-level factors associated with circulating concentrations of chondroitin sulfate and its subtypes are provided in Supplemental Figures 7 and 8. Intriguingly, 0S chondroitin sulfate concentrations at study entry were inversely correlated with an antecedent history of liver disease, potentially suggesting a hepatic source of this chondroitin sulfate subtype (Supplemental Figure 8A).

## Discussion

In this secondary analysis of the CLOVERS trial, we found that plasma chondroitin sulfate was both a prognostic and predictive biomarker in patients with sepsis-associated hypotension. Plasma chondroitin sulfate concentrations early in the course of sepsis care (with CLOVERS patient enrollment occurring a median of 61 minutes after study eligibility, defined as sepsis-induced hypotension despite 1 liter of crystalloid treatment) independently predicted 90-day mortality, similar to other circulating glycosaminoglycans (such as hyaluronic acid or heparan sulfate, ref. 7) more specific to endothelial glycocalyx degradation. Strikingly, plasma chondroitin sulfate concentrations at study enrollment predicted a patient’s subsequent response to a randomly assigned liberal or restrictive intravenous fluid resuscitation strategy, using 90-day mortality as the outcome. This heterogeneity of treatment effect was unique to chondroitin sulfate and was not observed with hyaluronic acid (a canonical glycosaminoglycan marker of endothelial glycocalyx shedding) or IL-6 (a canonical marker of systemic inflammation). These findings suggest that plasma chondroitin sulfate levels could inform decision-making between different fluid resuscitation strategies in patients with sepsis-induced hypotension. Indeed, on adjusted analysis the “inflection point” of plasma chondroitin sulfate at which the benefits of liberal versus restrictive fluid resuscitation flip is the 62nd percentile of patients, suggesting that the relevance of this predictive biomarker is not limited to a small subset of our cohort but rather applies to large numbers of patients with sepsis.

The mechanisms underlying the predictive capabilities of circulating chondroitin sulfate are uncertain. Unlike the glycosaminoglycans heparan sulfate and hyaluronic acid, which are anchored within the endothelial glycocalyx and do not circulate at high concentrations under healthy conditions, plasma chondroitin sulfate is abundant in normal blood (12, 17). Indeed, our findings are consistent with chondroitin sulfate not being simply a marker of endothelial glycocalyx degradation, as chondroitin sulfate was only moderately associated with traditional biomarkers of endothelial glycocalyx degradation such as heparan sulfate, hyaluronic acid, or the glycocalyx proteoglycan syndecan-1. Our findings also suggest that chondroitin sulfate is not simply a proxy for systemic inflammation, as circulating chondroitin sulfate level was minimally correlated with the canonical inflammatory marker IL-6. While the predictive capabilities of chondroitin sulfate persisted even after controlling for Sequential Organ Failure Assessment (SOFA) score (which includes measures of renal function) and preexisting chronic kidney disease, it is possible that plasma chondroitin sulfate may be a biomarker of early, subclinical renal injury. As patients with renal disease demonstrated a nonsignificant trend toward worsened survival with liberal fluid resuscitation in the parent CLOVERS study (2), an association of high chondroitin sulfate levels with early, subclinical renal failure could potentially explain the predictive capabilities of chondroitin sulfate observed in our study.

We additionally considered that the heterogeneity of treatment effect associated with chondroitin sulfate may relate to its role as a component of circulating proteoglycans such as bikunin, a constituent of inter-α-trypsin inhibitor (18). Inter-α-trypsin inhibitors are protective antiproteases that inhibit serine proteases such as leukocyte elastase and cathepsin G (19). In sepsis, inter-α-trypsin inhibitor is proteolytically cleaved, increasing the circulating concentrations of free, chondroitin sulfate-decorated bikunin (20). As bikunin enriched from the plasma of patients with septic shock was sufficient to induce endothelial damage ex vivo (21), an increase in circulating chondroitin sulfate could indicate state of endothelial injury in which pathogenic edema might be worsened by a liberal fluid resuscitation approach. However, we found that the differential responses to the fluid resuscitation strategy were largely associated with unsulfated chondroitin sulfate (Figure 5, A and B), as opposed to the 4-O sulfated chondroitin sulfates typically bound to bikunin (Figure 5, C and D) (12, 13). This finding suggests that any ability of circulating chondroitin sulfate to modify the response to fluid resuscitation is unrelated to bikunin-associated chondroitin sulfate. Alternatively, our results may represent a potential shift in chondroitin sulfate sulfation in sepsis (22, 23) or the exposure and circulation of cryptic undersulfated chondroitin sulfate sites by bikunin/inter-α-trypsin inhibitor fragmentation during sepsis (20).

Collectively, our findings identify an important yet unexplored line of mechanistic investigation in sepsis: how chondroitin sulfate modifies fluid resuscitation strategies. The lack of effect of randomized resuscitation strategy on plasma chondroitin sulfate measured at 24 hours (i.e., at the conclusion of protocolized resuscitation) or at 72 hours (Figure 3 and Supplemental Figure 4) suggests that increased plasma concentrations of chondroitin sulfate, while able to identify patient subtypes that are differentially responsive to fluid resuscitation strategy, are not part of the casual pathway of that differential responsiveness. However, this observation requires exploration with additional mechanistic investigation.

Regardless of its underlying biological mechanism, the predictive capabilities of plasma chondroitin sulfate in CLOVERS suggest a role for this biomarker in a precision medicine approach to sepsis resuscitation. Our findings (Figure 4, A and B) suggest that patients with high concentrations of plasma chondroitin sulfate at sepsis presentation would benefit from assignment to a restrictive intravenous fluid resuscitation strategy. However, our findings were derived using state-of-the-art mass spectrometry approaches that are expensive and infeasible for rapid implementation at the bedside. Future trials testing the value of plasma chondroitin sulfate in a sepsis treatment algorithm should therefore employ rapid, inexpensive point-of-care assays of circulating glycosaminoglycans. As circulating chondroitin sulfate arises from multiple nonglycocalyx sources, endothelial glycocalyx degradation markers such as plasma syndecan-1 (Figure 1) or sublingual intravital microscopy (4) are unlikely to serve as accurate proxies of plasma chondroitin sulfate. Future studies should test the bedside validity of direct assays of circulating glycosaminoglycans (such as dimethylmethylene blue [DMMB], ref. 9) as rapid indices of plasma chondroitin sulfate in patients with sepsis. It is unclear, however, if these assays (which typically target sulfated regions of glycosaminoglycans) will capture the heterogeneity of treatment effect observed in CLOVERS, which was largely driven by unsulfated chondroitin sulfate (Figure 5, A and B).

Our work provides additional insights into the mechanisms and consequences of endothelial glycocalyx shedding during critical illness. Previous preclinical studies have suggested that endothelial glycocalyx sheddases are induced by inflammatory stimuli (24, 25). However, our study only found a weak association between circulating IL-6 and biomarkers of endothelial glycocalyx shedding (heparan sulfate, hyaluronic acid, or syndecan-1) or circulating chondroitin sulfate. This finding suggests that circulating glycosaminoglycans are not simply a proxy for systemic inflammation, but likely represent distinct pathological processes such as vascular injury (potentially mediated by angiopoietin-2 signaling, ref. 26) or, for chondroitin sulfate, alterations in processes mediated by inter-α-trypsin inhibitor and/or bikunin signaling (18). Importantly, neither baseline hyaluronic acid nor IL-6 (or heparan sulfate and syndecan-1, as previously reported, ref. 7) were associated with differential responses to a randomized fluid resuscitation strategy, suggesting that the presence (and magnitude) of endothelial glycocalyx injury and/or systemic inflammation did not determine an individual patient’s subsequent response to a liberal or restrictive approach to intravenous fluids. Finally, our study suggests that sepsis-induced endothelial glycocalyx degradation is not simply the consequence of a single “sheddase.” In contrast to heparan sulfate, which is anchored to the endothelial surface by heparan sulfate proteoglycans such as syndecans, hyaluronic acid intercalates throughout the endothelial glycocalyx and interacts with the endothelial surface via cell membrane hyaladherins such as CD44 (4). Thus, hyaluronic acid shedding is unlikely to occur via heparan sulfate-specific sheddases (such as heparanase) previously implicated in septic endothelial glycocalyx degradation (4). The similar associations of plasma hyaluronic acid, heparan sulfate, and syndecan-1 — each of which is shed from the endothelial glycocalyx by distinct sheddases — with sepsis outcomes suggest the presence of coordinated, possibly redundant processes of endothelial glycocalyx shedding during human sepsis.

Our investigation has several limitations. As noted above, the biological mechanism responsible for the striking finding of heterogeneity of treatment effect for plasma chondroitin sulfate and intravenous fluid resuscitation strategy remains unknown. Further mechanistic investigation is needed to study the relative effect of plasma chondroitin sulfate and/or its associated proteins on the response to intravenous fluids. The second limitation regards our weighted analyses: although we controlled for numerous variables that could confound our results, the possibility of residual confounding remains. Finally, this study did not stratify patients by the type of intravenous fluid received. While the majority of patients were given balanced crystalloids for resuscitation, some received normal saline, and this variation was not controlled for in our analyses or original CLOVERS study (2).

In conclusion, this investigation identifies that, in patients with sepsis-induced hypotension, baseline chondroitin sulfate levels reflect pathobiological processes other than endothelial glycocalyx degradation and/or systemic inflammation. Strikingly, baseline chondroitin sulfate concentrations predict an individual’s response (as judged by the outcome of 90-day mortality) to a subsequent liberal versus restrictive fluid resuscitation strategy. This finding represents a major advance toward precision medicine approaches to intravenous fluid resuscitation in sepsis. Finally, this study validates chondroitin sulfate, hyaluronic acid, and IL-6 as prognostic biomarkers for sepsis mortality.

## Methods

### Sex as a biological variable.

The study cohort included both male and female patients with sepsis-associated hypotension. Inverse probability of selection weighting and generalized raking estimation strategies were used to ensure that this cohort reflected the entire CLOVERS population (Table 1 and Supplemental Tables 1 and 2).

### Analyses of circulating biomarkers.

We measured chondroitin sulfate and hyaluronic acid disaccharides (Supplemental Table 3A) with ultra high–performance liquid chromatography–mass spectrometry (UPLC-MS/MS) as described previously (7) with modifications. Briefly, we spiked all samples with C^13^-labeled heparan sulfate polysaccharide recovery calibrant (1 ng) prior to sample processing to later verify complete digestion of glycosaminoglycans and to account for any substantial sample loss during processing. We desalted 50 μL plasma using 3 kDa molecular weight cut-off column (Millipore, UFC500396) and performed overnight on-column digestion with heparinase I, II, III, and chondroitinase ABC. We removed enzymes with centrifugation and collected digested glycosaminoglycans. To quantify chondroitin sulfate and hyaluronic acid, we spiked in C^13^-labeled disaccharide calibrants, 1 ng each, (Supplemental Table 3B) and lyophilized samples. We then derivatized disaccharides with 2-aminoacridone (AMAC) in DMSO/glacial acetic acid (17:3, v/v) followed by addition of aqueous sodium cyanoborohydride. The reaction mixture was incubated at 45°C for 2 hours. We cleaned samples with C18 resin, washed with 0.1% formic acid solution, and eluted with 80% acetonitrile and 0.1% formic acid solution. We removed the organics with a speed vacuum concentrator overnight and reconstituted the samples with mobile phase A.

We quantified AMAC-derivatized disaccharides with an UltiMate 3000 LC system (Thermo) in tandem with QTRAP 5500 mass spectrometry (AB Sciex). Chromatography was reverse-phase and performed with Aquity UPLC BEH-C18 (Waters, 150 × 1.0, 1.7 μm) and with Aquity UPLC BEH-C18 guard column (Waters, 5 × 2.1, 1.7 μm). Disaccharides were separated using mobile phase A (95:5 water/methanol, 1 mM ammonium acetate, pH 8.6) and mobile phase B (95:5 methanol/ water, 1 mM ammonium acetate), with the gradient as follows: 0–15 minutes, 0%–15% B; 15–17 minutes, 15%–35% B; 17.01 minutes, 100% B; 20 minutes, 100% B; 20.01 minutes, 0% B; 23 minutes, 0% B, at a flow rate of 0.1 mL/min. The column temperature was held at 45°C. The mass spectrometer was operated in electrospray negative ionization mode with multiple reaction monitoring. Ionization parameters common to all analytes were set as follows: curtain gas at 30 psi, nebulizing gas at 40 psi, drying gas at 20 psi, ion spray voltage at –4,500 V, and source temperature at 400°C. Compound-specific parameters (entrance potential, declustering potential, collision energy, collision cell exit potential) are listed on Supplemental Table 3C. Dwell time for each analyte was 20 msec. The mass spectrometry data were acquired with Analyst 1.6.2. We determined the concentration of each analyte based on the internal calibrant as shown in Supplemental Table 3B.

Total chondroitin sulfate concentration within an individual sample was calculated as the sum of all 8 possible chondroitin sulfate disaccharides (Supplemental Table 3A) within that sample. In rare cases, select peaks for individual disaccharides were unable to be measured within a sample. In this case, the concentration of the missing disaccharide was imputed using information from the other, nonmissing disaccharides within that sample. At baseline, of the 574 × 8 = 4,592 disaccharide measurements, 6 (0.1%) of them were missing and were singly imputed prior to calculating the baseline total chondroitin sulfate value. At 24 hours after randomization, of the 476 × 8 = 3,808 constituents, 4 (0.1%) of them were missing and were singly imputed prior to calculating the 24-hour total chondroitin sulfate value.

Plasma IL-6 was measured using Human Luminex Discovery Assays (R&D Systems).

### Statistics.

We summarized the baseline and demographic characteristics of the study sample for chondroitin sulfate and hyaluronic acid, the study sample for IL-6, and the full CLOVERS study cohort with frequencies and percentages for discrete variables and with the median and interquartile range for quantitative variables. We used violin plots to display the distributions at baseline and at 24 hours (chondroitin sulfate and hyaluronic acid only) and 72 hours after study enrollment for those who did and did not die during the 90-day follow-up period and for those assigned to liberal or restrictive fluid strategy.

Because our study included nonrepresentative, stratified sampling from the original CLOVERS cohort, we conducted analyses using inverse probability of being sampled weighting (IPSW) (27) and generalized raking approaches (28, 29). IPSW weights each participant selected for plasma collection by the inverse of their probability of being sampled, while generalized raking further calibrates the sampling weights using information observed in the full CLOVERS cohort. Importantly, even though the substudy samples are not representative of the original CLOVERS cohort, by reweighting individuals selected for plasma collection, our findings generalize to the population represented by the original CLOVERS cohort (30). See the supplemental materials for more detail on the IPSW and generalized raking analysis strategies.

We applied IPSW with estimated weights for correlation estimates and tests and for Kaplan-Meier analyses. Sampling probabilities used to calculate the IPSW weights for chondroitin sulfate and hyaluronic acid are shown in Supplemental Table 4, and sampling probabilities for IL-6 are shown in Supplemental Table 5. The Kaplan-Meier analyses examined the unadjusted association between baseline biomarker tertiles and survival from randomization to 90 days after randomization.

For all regression models, we sought to adjust for a prespecified set of baseline covariates that might confound associations with mortality. This set includes randomized treatment assignment, baseline SOFA score, sex, age, race and ethnicity, ARDS at randomization, history of diabetes, history of heart failure, and history of end-stage renal disease. SOFA score and age were entered into the models using restricted cubic splines with 3 knots to permit nonlinear associations. After an initial model fit, the proportional hazards assumption was observed to be violated for associations with sex, heart failure history, and SOFA score. To address this concern, to avoid the uncertainty associated with making multiple decisions about proportional hazards assumptions across models, and to flexibly adjust for key potential confounders, we stratified all Cox model analyses by sex and heart failure history, and we included SOFA score interactions with follow-up time (0–4 days, 5–11 days, and 12–90 days) (7). Biomarker measurements were centered in all regression models. Because patient data were collected at 51 sites in the CLOVERS study, we used design-adjusted robust standard errors to account for clustering by site and to acknowledge the stratified sampling design (30). We combined sites with less or equal to 5 participants into 1 group with *N* = 23 participants to have sufficient data for each site.

We fit Cox proportional hazard regression models to examine (a) the baseline-covariate-adjusted biomarker associations with mortality through 90 days and (b) the extent to which the treatment (restrictive versus liberal resuscitation strategy) effect on mortality through 90 days varied according to baseline biomarker concentrations. These models controlled for the baseline covariates mentioned above. To fit the latter model, we added the randomized treatment assignment by biomarker concentration interaction to the former model. To adjust for the nonrepresentative sample, we used generalized raking.

To examine the randomized treatment assignment effect on biomarker concentrations at 24 hours (for chondroitin sulfate and hyaluronic acid) and the 72 hours (for IL-6), we performed linear regression after adjusting for the nonrepresentative sample with IPSW. These models controlled for baseline covariates mentioned above as well as the baseline biomarker concentration.

For all baseline models, there were 574 participants analyzed for chondroitin sulfate and hyaluronic acid and 1,369 for IL-6. For models with 24-hour (for chondroitin sulfate and hyaluronic acid) or 72-hour data (for IL-6), 476 participants were analyzed for chondroitin sulfate and hyaluronic acid and 844 participants for IL-6. We summarized model results graphically.

Additional statistical details are provided in the Supplemental Methods. All analyses were performed using R version 4.4.1, and analyses were performed with the survey, survival, Hmisc, and packages (31–38). A *P* value less than 0.05 was considered significant.

### Study approval.

The CLOVERS study and this ancillary analysis were approved by Vanderbilt University Medical Center IRB, which acted as a central IRB, and patients were enrolled using a written informed consent, as previously described (2).

### Data availability.

Data underlying all dot plot, survival analyses, and violin figures are provided in the accompanying Supporting Data Values file. Details on the code used to fit the regression models are provided in the Supplemental R Code for Model Fitting. Deidentified human data are available upon reasonable request to the corresponding author (EPS).

## Author contributions

KO, CJL, JSS, NIS, and EPS conceived and designed the study. KO, SAM, IN, DS, AI, KCH, AD, CL, AR, and MAM acquired and analyzed the data. BY, GA, RT, CDG, RCB, YN, CJL, and JSS performed statistical analyses. KO, MSK, BY, ISD, WHS, JSS, NIS, and EPS drafted the manuscript. All authors contributed to the critical review and interpretation of the data and manuscript and intellectual content. KO is listed as the first coauthor, as she directly contributed to the conception and scope of the project.

## Funding support

This work is the result of NIH funding, in whole or in part, and is subject to the NIH Public Access Policy. Through acceptance of this federal funding, the NIH has been given a right to make the work publicly available in PubMed Central.

- NIH grants (R01HL149422 to NIS and EPS, R01HL094786 to JSS).

## Supplementary Material

Supplemental data

ICMJE disclosure forms

Supporting data values

## Figures and Tables

**Table 1:**
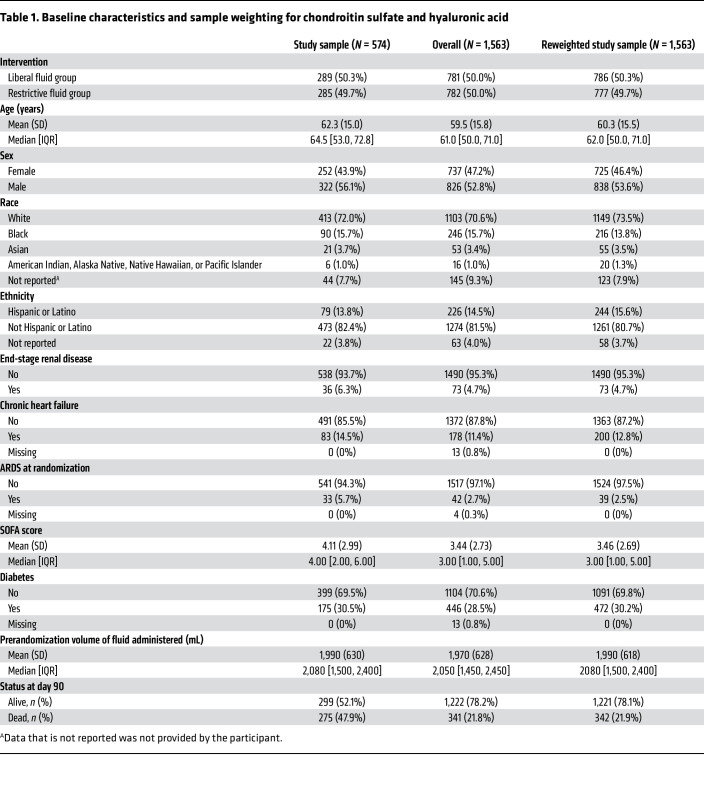
Baseline characteristics and sample weighting for chondroitin sulfate and hyaluronic acid

## Version 1 02/03/2026

In-Press Preview

## Version 2 04/01/2026

Electronic publication
