# Supplementary material for: Plasma chondroitin sulfate predicts the effectiveness of fluid resuscitation strategies in patients with sepsis
Source: J Clin Invest. 2026 Feb 3;136(7):e202480. doi: 10.1172/JCI202480 (PMC13038195; doi:10.1172/JCI202480)
Supplement: Supplemental data [file jci-136-202480-s087.pdf]

**Supplemental Materials**

Plasma chondroitin sulfate predicts the effectiveness of fluid resuscitation strategies in patients with sepsis

*Kaori Oshima[1], *Bailu Yan[2], Ran Tao[2,3], Gustavo Amorim[2], Chiara Di Gravio[4], Sarah A. McMurtry[5], Ryan C. Burke[6], Yunbi Nam[2], Ina Nikolli[1], Max S. Kravitz[6], Daniel Stephenson[7], Aaron Issaian[7], Kirk C. Hansen[7], Angelo D'Alessandro[7], Ivor S. Douglas[5,8], Wesley H. Self[9], Christopher J. Lindsell[10], Carolyn Leroux[11], Angelika Ringor[11], Michael A. Matthay[11], Jonathan S. Schildcrout[2], †Nathan I. Shapiro[6], †Eric P. Schmidt[1]

*Contributed equally to the manuscript (first author)

†Contributed equally to the manuscript (last author)

**List of supplemental material**


Page 1: **Table of Contents**
Page 2-3: **Supplemental Statistical Methods**
Page 4-8: **Supplemental R Code for Model Fitting**
Page 9-10: **Supplemental Table 1:** Additional baseline characteristics and sample weighting for chondroitin sulfate and hyaluronic acid
Page 11-13: **Supplemental Table 2**: Baseline characteristics and sample weighting for IL-6
Page 14: **Supplemental Table 3**: Glycosaminoglycan structures, internal standards, and analytical parameters for mass spectrometry quantification
Page 15: **Supplemental Table 4**: Sampling probabilities used to calculate the inverse probability of selection weights in analyses of chondroitin sulfate and hyaluronic acid
Page 16: **Supplemental Table 5**: Sampling probabilities used to calculate the inverse probability of selection weights in analyses of IL-6
Page 17: **Supplemental Figure 1**. Baseline plasma chondroitin sulfate is an independent predictor of time to all-cause mortality within 90 days of CLOVERS randomization.
Page 18: **Supplemental Figure 2**. Baseline plasma hyaluronic acid is an independent predictor of time to all-cause mortality within 90 days of CLOVERS randomization.
Page 19: **Supplemental Figure 3**. Baseline plasma IL-6 is an independent predictor of time to all-cause mortality within 90 days of CLOVERS randomization
Page 20: **Supplemental Figure 4**. Randomization to a liberal or restrictive fluid-resuscitation approach has no impact on plasma chondroitin sulfate on multivariate analysis.
Page 21: **Supplemental Figure 5**. Randomization to a liberal or restrictive fluid-resuscitation approach has no impact on hyaluronic acid shedding on multivariate analysis
Page 22: **Supplemental Figure 6**. Randomization to a liberal or restrictive fluid-resuscitation approach has no impact on plasma IL-6 on multivariate analysis
Page 24-25: **Supplemental Figure 8.** Association of baseline plasma concentrations of chondroitin sulfate subtypes with patient characteristics.

## Supplemental Statistical Methods

For the Cox proportional hazard regression model, the hazard rate for subject $i$ is estimated using:

$$\lambda_i(t) = \lambda_0(t)\exp(\beta_x X_i + \beta_z Z_i),$$

where

- $i$: subject number
- $\lambda_0(t)$: baseline hazard function
- $X_i$: subject $i$'s expensive biomarker measurement that is only available in the phase-II sample
- $Z_i$: subject $i$'s other phase-I adjustment covariates
- $\beta_x$, $\beta_z$: regression coefficients for $X_i$ and $Z_i$
- $\lambda_i(t)$: subject $i$'s hazard at time $t$ given $X_i$ and $Z_i$

The **inverse probability of being sampled weighting (IPSW)** with estimated weights uses the inverse of the observed sampling probability of each subject and derives the coefficient estimates by solving a weighted score function[1]:

$$\sum_{i=1}^{N} \frac{R_i}{\pi_i} \frac{\partial log P(Y_i|X_i, Z_i; \beta)}{\partial \beta} = \sum_{i=1}^{N} R_i w_i U_i(\beta) = 0, with\ w_i = \frac{1}{\pi_i},$$

where

- $R_i$: binary variable, $R_i = 1$ indicates that subject $i$ is selected in the phase-II sample
- $Y_i$: outcome variable
- $X_i$: subject $i$'s expensive biomarker measurement that is only available in the phase-II sample
- $Z_i$: subject $i$'s other phase-I adjusted covariates
- $P(Y_i|X_i, Z_i; \beta)$: partial log-likelihood function for subject $i$ for the Cox model or the log-likelihood for subject $i$ for the linear regression model
- $U_i(\beta)$: the score equation for subject $i$ for the Cox model or the linear regression model
- $\pi_i$: sampling probability calculated using observed data for each sampling stratum
- $w_i$: weight of IPSW

This method is often called the Horvitz-Thompson (HT) method.

**Generalized raking (GR)** adjusts IPSW sampling weights based on auxiliary variable information, which gives a more efficient estimator than IPSW[2,3,4]. Suppose $H_i$ is the auxiliary variable and $d(.)$ is a distance measuring function.[2,3] The generalized raking estimator is solved by the score function:

$$\sum_{i=1}^{N} R_i \frac{g_i}{\pi_i} U_i(\beta) = \sum_{i=1}^{N} R_i w_{i,GR} U_i(\beta) = 0,$$

where

- $\beta$: regression coefficients
- $w_{i,GR} = \frac{g_i}{\pi_i}$: calibrated weights satisfying $\sum_{i=1}^{N} R_i \frac{g_i}{\pi_i} H_i = \sum_{i=1}^{N} H_i$ and minimizing the total discrepancy function for $w_i$ and $w_{i,GR}$ i.e., $\sum_{i=1}^{N} R_i d(\frac{1}{\pi_i}, \frac{g_i}{\pi_i})$.
- $H_i$: influence functions for $\beta$ estimates from the Cox model using imputed estimates of the phase-II data

This approach matches the weighted total of auxiliary variables in phase-II data to the observed phase-I population, so the estimator better represents the original, full CLOVERS cohort. Using the influence functions for generalized raking is expected to give more efficient estimates than using IPSW weights or weight calibration by raw auxiliary variables.[2,3,4]

**Supplemental Statistical References**


1. Horvitz DG, Thompson DJ. A Generalization of Sampling Without Replacement from a Finite Universe. *J Am Stat Assoc* 1952; **47**: 663–85.
2. Deville J-C, Särndal C-E. Calibration Estimators in Survey Sampling. *J Am Stat Assoc* 1992; **87**: 376–82.
3. Deville J-C, Särndal C-E, Sautory O. Generalized Raking Procedures in Survey Sampling. *J Am Stat Assoc* 1993; **88**: 1013–20.
4. White H. Maximum Likelihood Estimation of Misspecified Models. *Econometrica* 1982; **50**: 1.

**Supplemental R Code for Model Fitting**

**Variable Definitions for Coding**

ltotCS_1: Log of Baseline Chondroitin Sulfate (ng/ml)
lHA_1: Log of Baseline Hyaluronic Acid (ng/ml)
lIL6_1: Log of Baseline IL-6 (pg/ml)
lsynd_1: Log of Baseline Syndecan-1 (ng/ml)
ltotHS_1: Log of Baseline total Heparan Sulfate (ng/ml)
ltotCS_2: Log of 24 Hrs Chondroitin Sulfate (ng/ml)
lHA_2: Log of 24 Hrs Hyaluronic Acid (ng/ml)
lIL6_3: Log of 72 Hrs IL-6 (pg/ml)
rand_trt: Intervention (“Liberal Fluid Group” or “Restrictive Fluid Group”)
ards: ARDS at Randomization (“Yes” or “No”)
age: Age (years)
dmg_sex: Sex (“Male” or “Female”)
race_ethnicity: Race/Ethnicity (“Non-Hispanic White”, “Hispanic”, “Non-Hispanic Black”, “Not reported”, or “Non-Hispanic Other”)
hf: Chronic Heart Failure (“Yes” or “No”)
diabetes: Diabetes (“Yes” or “No”)
sofa: SOFA Score
kidney: End-stage Renal Disease (“Yes” or “No”)
time_group: indicator for periods of 0-4 days, 5-11 days, and 12-90 days of follow-up to address concerns about violations of the proportional hazards assumption and to flexibly adjust for potentially important confounder variables (“1” = “0-4 days”, “2” = “5-11 days”, “3” = “12-90 days”).
tstart: start time of survival analysis (-1, 4, or 11)
time_90: survival time until day 90
status_90: status at day 90 (“0” = “Alive”, “1” = “Dead”)
site: study site
CS1.tert: 3 tertile groups divided by baseline chondroitin sulfate
HA1.tert: 3 tertile groups divided by baseline hyaluronic acid
IL61.tert: 3 tertile groups divided by baseline IL-6
samp_probs_mass_spec_bl: baseline chondroitin sulfate and hyaluronic acid analyses sampling probability
samp_probs_mass_spec_bl_24: baseline and 24 Hrs chondroitin sulfate and hyaluronic acid analyses sampling probability
samp_probs_il6_bl: baseline IL-6 analysis sampling probability
samp_probs_il6_bl_72: baseline and 72 Hrs IL-6 analysis sampling probability

**R Code for model fits**

**Figure 2:**
Figure 2B:

survfit(Surv(time_90, status_90) ~ CS1.tert, weights = 1/samp_probs_mass_spec_bl, data =bl_sampled_no_missing)

Figure 2C, Supplemental Figure 1:

dd_desn <- twophase(id = list(~1, ~1), strata = list(NULL, ~site), weights = list(NULL, ~I(1/samp_probs_mass_spec_bl)), subset = ~mass_spec_bl_sampled==1, data = v.reduced.dat, method = 'simple')

rak_desn_cal <- survey::calibrate(dd_desn, formula = as.formula(paste("~ gr + ", paste(colnames(inffun_imp), collapse = " + "))), phase=2, calfun = "raking")
svycoxph(Surv(tstart,time_90, status_90) ~ rcs(I(ltotCS_1-8.979272), parms = c(-0.3935512, 0.0000000, 0.4397660)) + rand_trt + rcs(sofa, parms = c(1,3,8)) + rcs(sofa, parms = c(1,3,8)):strata(time_group) + rcs(age, parms = c(40.0, 64.0, 80.7)) + kidney + race_ethnicity + diabetes + ards + strata(dmg_sex) + strata(hf), design = rak_desn_cal)

Figure 2E:

survfit(Surv(time_90, status_90) ~ HA1.tert, weights = 1/samp_probs_mass_spec_bl, data =bl_sampled_no_missing)

Figure 2F, Supplemental Figure 2:

dd_desn <- twophase(id = list(~1, ~1), strata = list(NULL, ~site), weights = list(NULL, ~I(1/samp_probs_mass_spec_bl)), subset = ~mass_spec_bl_sampled==1, data = v.reduced.dat, method = 'simple')

rak_desn_cal <- survey::calibrate(dd_desn, formula = as.formula(paste("~ gr + ", paste(colnames(inffun_imp), collapse = " + "))), phase=2, calfun = "raking")
svycoxph(Surv(tstart,time_90, status_90) ~ rcs(I(lHA_1-5.116802), parms = c(-1.524523, 0.000000, 2.227362)) + rand_trt + rcs(sofa, parms = c(1,3,8)) + rcs(sofa, parms = c(1,3,8)):strata(time_group) + rcs(age, parms = c(40.0, 64.0, 80.7)) + kidney + race_ethnicity + diabetes + ards + strata(dmg_sex) + strata(hf), design = rak_desn_cal)

Figure 2H:

survfit(Surv(time_90, status_90) ~ IL61.tert, weights = 1/samp_probs_il6_bl, data =il6_wide_bl_no_missing)

Figure 2I, Supplemental Figure 3:

dd_desn <- twophase(id = list(~1, ~1), strata = list(NULL, ~site), weights = list(NULL, ~I(1/samp_probs_il6_bl)), subset = ~il6_bl_sampled==1, data = v.reduced.dat, method = 'simple')

rak_desn_cal <- survey::calibrate(dd_desn, formula = as.formula(paste("~ gr + ", paste(colnames(inffun_imp), collapse = " + "))), phase=2, calfun = "raking")
svycoxph(Surv(tstart,time_90, status_90) ~ rcs(I(lIL6_1-4.18496), parms = c(-2.087183, 0.000000, 3.700861)) + rand_trt + rcs(sofa, parms = c(1,3,8)) + rcs(sofa, parms = c(1,3,8)):strata(time_group) + rcs(age, parms = c(40.0, 64.0, 80.7)) + kidney + race_ethnicity + diabetes + ards + strata(dmg_sex) + strata(hf), design = rak_desn_cal)

**Figure 4:**

Figure 4A,B:

dd_desn <- twophase(id = list(~1, ~1), strata = list(NULL, ~site), weights = list(NULL, ~I(1/samp_probs_mass_spec_bl)), subset = ~mass_spec_bl_sampled==1, data = v.reduced.dat, method = 'simple')

rak_desn_cal <- survey::calibrate(dd_desn, formula = as.formula(paste("~ gr + ", paste(colnames(inffun_imp), collapse = " + "))), phase=2, calfun = "raking")
svycoxph(Surv(tstart,time_90, status_90) ~ rcs(I(ltotCS_1-8.979272), parms = c(-0.3935512, 0.0000000, 0.4397660)) * rand_trt + rcs(sofa, parms = c(1,3,8)) + rcs(sofa, parms = c(1,3,8)):strata(time_group) + rcs(age, parms = c(40.0, 64.0, 80.7)) + kidney + race_ethnicity + diabetes + ards + strata(dmg_sex) + strata(hf), design = rak_desn_cal)

Figure 4C,D:

dd_desn <- twophase(id = list(~1, ~1), strata = list(NULL, ~site), weights = list(NULL, ~I(1/samp_probs_mass_spec_bl)), subset = ~mass_spec_bl_sampled==1, data = v.reduced.dat, method = 'simple')

rak_desn_cal <- survey::calibrate(dd_desn, formula = as.formula(paste("~ gr + ", paste(colnames(inffun_imp), collapse = " + "))), phase=2, calfun = "raking")
svycoxph(Surv(tstart,time_90, status_90) ~ rcs(I(lHA_1-5.116802), parms = c(-1.524523, 0.000000, 2.227362)) * rand_trt + rcs(sofa, parms = c(1,3,8)) + rcs(sofa, parms = c(1,3,8)):strata(time_group) + rcs(age, parms = c(40.0, 64.0, 80.7)) + kidney + race_ethnicity + diabetes + ards + strata(dmg_sex) + strata(hf), design = rak_desn_cal)

Figure 4E,F:

dd_desn <- twophase(id = list(~1, ~1), strata = list(NULL, ~site), weights = list(NULL, ~I(1/samp_probs_il6_bl)), subset = ~il6_bl_sampled==1, data = v.reduced.dat, method = 'simple')

rak_desn_cal <- survey::calibrate(dd_desn, formula = as.formula(paste("~ gr + ", paste(colnames(inffun_imp), collapse = " + "))), phase=2, calfun = "raking")
svycoxph(Surv(tstart,time_90, status_90) ~ rcs(I(lIL6_1-4.18496), parms = c(-2.087183, 0.000000, 3.700861)) * rand_trt + rcs(sofa, parms = c(1,3,8)) + rcs(sofa, parms =

c(1,3,8)):strata(time_group) + rcs(age, parms = c(40.0, 64.0, 80.7)) + kidney + race_ethnicity + diabetes + ards + strata(dmg_sex) + strata(hf), design = rak_desn_cal)

**Supplemental Figure 4:**

dd_desn_ols_bl24 <- twophase(id = list(~1, ~1), strata = list(NULL, ~site), weights = list(NULL, ~I(1/samp_probs_mass_spec_bl_24)), subset = ~mass_spec_bl_24_sampled==1, data = all_bl_data_w_sampled, method = 'simple')
svyglm(ltotCS_2 ~ rcs(I(ltotCS_1-8.979272), parms = c(-0.3935512, 0.0000000, 0.4397660)) + rcs(age, parms = c(40.0, 64.0, 80.7)) + dmg_sex + rand_trt + race_ethnicity + diabetes + hf + kidney + rcs(sofa, parms = c(1,3,8)) + ards, design = dd_desn_ols_bl24, family = gaussian)

**Supplemental Figure 5:**

dd_desn_ols_bl24 <- twophase(id = list(~1, ~1), strata = list(NULL, ~site), weights = list(NULL, ~I(1/samp_probs_mass_spec_bl_24)), subset = ~mass_spec_bl_24_sampled==1, data = all_bl_data_w_sampled, method = 'simple')
svyglm(lHA_2 ~ rcs(I(lHA_1-5.116802), parms = c(-1.524523, 0.000000, 2.227362)) + rcs(age, parms = c(40.0, 64.0, 80.7)) + dmg_sex + rand_trt + race_ethnicity + diabetes + hf + kidney + rcs(sofa, parms = c(1,3,8)) + ards, design = dd_desn_ols_bl24, family = gaussian)

**Supplemental Figure 6:**

dd_desn_ols_bl72 <- twophase(id = list(~1, ~1), strata = list(NULL, ~site), weights = list(NULL, ~I(1/samp_probs_il6_bl_72)), subset = ~il6_bl_72_sampled==1, data = all_bl_data_w_sampled, method = 'simple')
svyglm(lIL6_3 ~ rcs(I(lIL6_1-4.18496), parms = c(-2.087183, 0.000000, 3.700861)) + rcs(age, parms = c(40.0, 64.0, 80.7)) + dmg_sex + rand_trt + race_ethnicity + diabetes + hf + kidney + rcs(sofa, parms = c(1,3,8)) + ards, design = dd_desn_ols_bl72, family = gaussian)

**Supplemental Figure 7:**
dd_desn_ols_bl <- twophase(id = list(~1, ~1), strata = list(NULL, ~site), weights = list(NULL, ~I(1/samp_probs_mass_spec_bl)), subset = ~mass_spec_bl_sampled==1, data = bl_dat_w_liver, method = 'simple')
mod_ipw_ols_bl <- svyglm(ltotCS_1 ~ rcs(age, parms = c(40.0, 64.0, 80.7)) + dmg_sex + rand_trt + race_ethnicity + diabetes + hf + kidney + rcs(sofa, parms = c(1,3,8)) + ards + charl_liver, design = dd_desn_ols_bl, family = gaussian)

**Supplemental Figure 8:**
dd_desn_ols_bl <- twophase(id = list(~1, ~1), strata = list(NULL, ~site), weights = list(NULL, ~I(1/samp_probs_mass_spec_bl)), subset = ~mass_spec_bl_sampled==1, data = bl_dat_w_liver, method = 'simple')

Supplemental Figure 8A:
mod_ipw_ols_bl_cs0s <- svyglm(ICS0S_1 ~ rcs(age, parms = c(40.0, 64.0, 80.7)) + dmg_sex + rand_trt + race_ethnicity + diabetes + hf + kidney + rcs(sofa, parms = c(1,3,8)) + ards + charl_liver,  design = dd_desn_ols_bl, family = gaussian)

Supplemental Figure 8B:
mod_ipw_ols_bl_cs4s <- svyglm(ICS4S_1 ~ rcs(age, parms = c(40.0, 64.0, 80.7)) + dmg_sex + rand_trt + race_ethnicity + diabetes + hf + kidney + rcs(sofa, parms = c(1,3,8)) + ards + charl_liver,  design = dd_desn_ols_bl, family = gaussian)

Supplemental Figure 8C:
mod_ipw_ols_bl_cs6s <- svyglm(ICS6S_1 ~ rcs(age, parms = c(40.0, 64.0, 80.7)) + dmg_sex + rand_trt + race_ethnicity + diabetes + hf + kidney + rcs(sofa, parms = c(1,3,8)) + ards + charl_liver,  design = dd_desn_ols_bl, family = gaussian)

**Supplemental Table 1**: Additional baseline characteristics and sample weighting for chondroitin sulfate and hyaluronic acid

| | Study Sample (N=574) | Overall (N=1563) | Reweighted Study Sample (N=1563) |
|---|---|---|---|
| **Location prior to Hospitalization** | | | |
| ED | 522 (90.9%) | 1437 (91.9%) | 1458 (93.3%) |
| ICU | 49 (8.5%) | 106 (6.8%) | 95 (6.1%) |
| Other | 3 (0.5%) | 20 (1.3%) | 9 (0.6%) |
| **Systolic Blood Pressure (mm Hg)** | | | |
| Mean (SD) | 92.9 (12.0) | 93.5 (12.1) | 92.8 (10.9) |
| Median [IQR] | 93.0 [86.0, 98.0] | 93.0 [87.0, 98.0] | 93.0 [87.0, 98.0] |
| **Time from Meeting Trial Eligibility Criteria to Randomization (min)** | | | |
| Mean (SD) | 77.9 (63.5) | 79.1 (67.4) | 74.5 (60.9) |
| Median [IQR] | 60.0 [26.3, 114] | 61.0 [26.0, 116] | 58.0 [25.0, 110] |
| **Most Common Primary Sources of Infection** | | | |
| Pneumonia | 184 (32.1%) | 417 (26.7%) | 445 (28.5%) |
| Urinary tract infection | 95 (16.6%) | 320 (20.5%) | 308 (19.7%) |
| Skin or soft-tissue infection | 57 (9.9%) | 179 (11.5%) | 166 (10.6%) |
| Intra-abdominal infection | 70 (12.2%) | 146 (9.3%) | 175 (11.2%) |
| Vascular catheter-related infection | 5 (0.9%) | 16 (1.0%) | 13 (0.8%) |
| Endocarditis or endovascular infection | 4 (0.7%) | 17 (1.1%) | 11 (0.7%) |
| Central nervous system infection | 3 (0.5%) | 5 (0.3%) | 6 (0.4%) |
| Other source of infection | 23 (4.0%) | 92 (5.9%) | 66 (4.2%) |
| Flu/other virus confirmed by testing | 4 (0.7%) | 17 (1.1%) | 11 (0.7%) |
| COVID-19 confirmed by testing | 0 (0%) | 5 (0.3%) | 0 (0%) |
| Unknown | 129 (22.5%) | 349 (22.3%) | 363 (23.2%) |
| **Pre-Randomization Invasive Mechanical Ventilation** | | | |

| | | | |
|---|---|---|---|
| No | 383 (66.7%) | 1216 (77.8%) | 1211 (77.5%) |
| Yes | 191 (33.3%) | 341 (21.8%) | 352 (22.5%) |
| Missing | 0 (0%) | 6 (0.4%) | 0 (0%) |
| **WBC Count (1000/mm3)** | | | |
| Mean (SD) | 13900 (10900) | 13800 (10900) | 13400 (9700) |
| Median [IQR] | 12200 [7000, 18000] | 12100 [7000, 17800] | 11800 [7100, 17700] |
| Missing | 4 (0.7%) | 11 (0.7%) | 11 (0.7%) |
| **Creatinine (mg/dL)** | | | |
| Mean (SD) | 2.07 (1.92) | 1.90 (1.82) | 1.93 (1.97) |
| Median [IQR] | 1.50 [0.900, 2.40] | 1.30 [0.860, 2.15] | 1.30 [0.850, 2.20] |
| Missing | 5 (0.9%) | 22 (1.4%) | 13 (0.8%) |
| **Lactate (mmol/L)** | | | |
| Mean (SD) | 3.36 (2.86) | 2.93 (2.48) | 2.99 (2.51) |
| Median [IQR] | 2.30 [1.60, 4.10] | 2.20 [1.50, 3.40] | 2.20 [1.50, 3.50] |
| Missing | 62 (10.8%) | 173 (11.1%) | 156 (10.0%) |

**Supplemental Table 2: Baseline characteristics and sample weighting for IL-6**

| | **Study Sample (N=1369)** | **CLOVERS Cohort (N=1563)** | **Reweighted Study Sample (N=1563)** |
|---|---|---|---|
| **Intervention** | | | |
| Liberal Fluid Group | 687 (50.2%) | 781 (50.0%) | 785 (50.2%) |
| Restrictive Fluid Group | 682 (49.8%) | 782 (50.0%) | 778 (49.8%) |
| **Age (years)** | | | |
| Mean (SD) | 60.0 (15.8) | 59.5 (15.8) | 60.0 (15.7) |
| Median [IQR] | 62.0 [50.0, 71.0] | 61.0 [50.0, 71.0] | 62.0 [50.0, 71.0] |
| **Sex** | | | |
| Female | 641 (46.8%) | 737 (47.2%) | 731 (46.8%) |
| Male | 728 (53.2%) | 826 (52.8%) | 832 (53.2%) |
| **Race** | | | |
| White | 966 (70.6%) | 1103 (70.6%) | 1102 (70.5%) |
| Black | 220 (16.1%) | 246 (15.7%) | 252 (16.1%) |
| Asian | 46 (3.4%) | 53 (3.4%) | 53 (3.4%) |
| American Indian, Alaska Native, Native Hawaiian, or Pacific Islander | 11 (0.8%) | 16 (1.0%) | 13 (0.8%) |
| Not Reported | 126 (9.2%) | 145 (9.3%) | 144 (9.2%) |
| **Ethnicity** | | | |
| Hispanic or Latino | 207 (15.1%) | 226 (14.5%) | 236 (15.1%) |
| Not Hispanic or Latino | 1110 (81.1%) | 1274 (81.5%) | 1268 (81.1%) |
| Not reported | 52 (3.8%) | 63 (4.0%) | 59 (3.8%) |
| **Location prior to Hospitalization** | | | |
| ED | 1261 (92.1%) | 1437 (91.9%) | 1440 (92.1%) |
| ICU | 95 (6.9%) | 106 (6.8%) | 108 (6.9%) |
| Other | 13 (0.9%) | 20 (1.3%) | 16 (1.0%) |
| **End-stage Renal Disease** | | | |
| No | 1304 (95.3%) | 1490 (95.3%) | 1488 (95.2%) |
| Yes | 65 (4.7%) | 73 (4.7%) | 75 (4.8%) |
| **Chronic Heart Failure** | | | |
| No | 1193 (87.1%) | 1372 (87.8%) | 1361 (87.1%) |
| Yes | 164 (12.0%) | 178 (11.4%) | 188 (12.0%) |
| Missing | 12 (0.9%) | 13 (0.8%) | 14 (0.9%) |
| **ARDS at Randomization** | | | |
| No | 1332 (97.3%) | 1517 (97.1%) | 1522 (97.4%) |
| Yes | 37 (2.7%) | 42 (2.7%) | 41 (2.6%) |
| Missing | 0 (0%) | 4 (0.3%) | 0 (0%) |
| **SOFA Score** | | | |

| | | | |
|---|---|---|---|
| Mean (SD) | 3.51 (2.72) | 3.44 (2.73) | 3.51 (2.72) |
| Median [IQR] | 3.00 [1.00, 5.00] | 3.00 [1.00, 5.00] | 3.00 [1.00, 5.00] |
| **Diabetes** | | | |
| No | 959 (70.1%) | 1104 (70.6%) | 1094 (70.0%) |
| Yes | 398 (29.1%) | 446 (28.5%) | 455 (29.1%) |
| Missing | 12 (0.9%) | 13 (0.8%) | 14 (0.9%) |
| **Systolic Blood Pressure (mm Hg)** | | | |
| Mean (SD) | 93.4 (12.2) | 93.5 (12.1) | 93.4 (12.2) |
| Median [IQR] | 93.0 [86.0, 98.0] | 93.0 [87.0, 98.0] | 93.0 [86.0, 98.0] |
| **Time from Meeting Trial Eligibility Criteria to Randomization (min)** | | | |
| Mean (SD) | 79.2 (65.1) | 79.1 (67.4) | 79.2 (65.1) |
| Median [IQR] | 62.0 [26.0, 118] | 61.0 [26.0, 116] | 62.0 [26.0, 118] |
| **Pre-Randomization Volume of Fluid Administered (mL)** | | | |
| Mean (SD) | 1980 (629) | 1970 (628) | 1980 (629) |
| Median [IQR] | 2050 [1500, 2500] | 2050 [1450, 2450] | 2050 [1500, 2500] |
| **Most Common Primary Sources of Infection** | | | |
| Pneumonia | 375 (27.4%) | 417 (26.7%) | 427 (27.3%) |
| Urinary tract infection | 289 (21.1%) | 320 (20.5%) | 330 (21.1%) |
| Skin or soft-tissue infection | 145 (10.6%) | 179 (11.5%) | 166 (10.6%) |
| Intra-abdominal infection | 129 (9.4%) | 146 (9.3%) | 148 (9.5%) |
| Vascular catheter-related infection | 15 (1.1%) | 16 (1.0%) | 17 (1.1%) |
| Endocarditis or endovascular infection | 15 (1.1%) | 17 (1.1%) | 17 (1.1%) |
| Central nervous system infection | 5 (0.4%) | 5 (0.3%) | 6 (0.4%) |
| Other source of infection | 71 (5.2%) | 92 (5.9%) | 81 (5.2%) |
| Flu/other virus confirmed by testing | 15 (1.1%) | 17 (1.1%) | 17 (1.1%) |
| COVID-19 confirmed by testing | 5 (0.4%) | 5 (0.3%) | 6 (0.4%) |
| Unknown | 305 (22.3%) | 349 (22.3%) | 349 (22.3%) |
| **Pre-Randomization Invasive Mechanical Ventilation** | | | |
| No | 1064 (77.7%) | 1216 (77.8%) | 1216 (77.8%) |
| Yes | 305 (22.3%) | 341 (21.8%) | 347 (22.2%) |

| | | | |
|---|---|---|---|
| Missing | 0 (0%) | 6 (0.4%) | 0 (0%) |
| **WBC Count (1000/mm3)** | | | |
| Mean (SD) | 14000 (11200) | 13800 (10900) | 14000 (11200) |
| Median [IQR] | 12200 [7000, 17900] | 12100 [7000, 17800] | 12200 [7000, 17900] |
| Missing | 10 (0.7%) | 11 (0.7%) | 11 (0.7%) |
| **Creatinine (mg/dL)** | | | |
| Mean (SD) | 1.95 (1.87) | 1.90 (1.82) | 1.95 (1.87) |
| Median [IQR] | 1.33 [0.870, 2.20] | 1.30 [0.860, 2.15] | 1.33 [0.870, 2.20] |
| Missing | 17 (1.2%) | 22 (1.4%) | 20 (1.3%) |
| **Lactate (mmol/L)** | | | |
| Mean (SD) | 2.93 (2.41) | 2.93 (2.48) | 2.93 (2.42) |
| Median [IQR] | 2.20 [1.50, 3.50] | 2.20 [1.50, 3.40] | 2.20 [1.50, 3.50] |
| Missing | 138 (10.1%) | 173 (11.1%) | 158 (10.1%) |
| **Status at Day 90** | | | |
| Alive | 1075 (78.5%) | 1222 (78.2%) | 1221 (78.1%) |
| Dead | 294 (21.5%) | 341 (21.8%) | 342 (21.9%) |

**Supplemental Table 3: Glycosaminoglycan structures, internal standards, and analytical parameters for mass spectrometry quantification**
(A) Disaccharide structures of chondroitin sulfate, hyaluronic acid, and heparan sulfate (used for internal standards). As hyaluronic acid is unsulfated, all disaccharides are identical. (B) C13 internal calibrants for each disaccharide analyte. (C) Compound-specific parameters for mass spectrometry MS/MS.

(A)

Chondroitin Sulfate (CS)

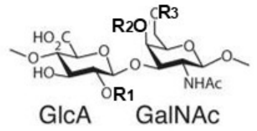


Hyaluronic Acid (HA)

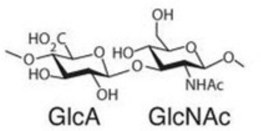


Heparan Sulfate (HS)

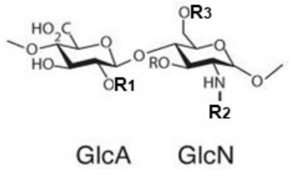


| | R1 (2-O) | R2 (4-O) | R3 (6-O) |
|---|---|---|---|
| ΔCS-0S | -H | -H | -H |
| ΔCS-2S | -SO3H | -H | -H |
| ΔCS-4S | -H | -SO3H | -H |
| ΔCS-6S | -H | -H | -SO3H |
| ΔCS-2S4S | -SO3H | -SO3H | -H |
| ΔCS-4S6S | -H | -SO3H | -SO3H |
| ΔCS-2S6S | -SO3H | -H | -SO3H |
| ΔCS-2S4S6S | -SO3H | -SO3H | -SO3H |

| | R1 (2-O) | R2 (N) | R3 (6-O) |
|---|---|---|---|
| ΔHS-0S | -H | -Ac | -H |
| ΔHS-2S | -SO3H | -Ac | -H |
| ΔHS-NS | -H | -SO3H | -H |
| ΔHS-6S | -H | -Ac | -SO3H |
| ΔHS-2SNS | -SO3H | -SO3H | -H |
| ΔHS-NS6S | -H | -SO3H | -SO3H |
| ΔHS-2S6S | -SO3H | -Ac | -SO3H |
| ΔHS-2SNS6S | -SO3H | -SO3H | -SO3H |

(B)

| Disaccharide Analyte | C13 Labeled Internal Calibrant |
|---|---|
| ΔCS-0S | ΔC13-CS-0S |
| ΔCS-2S | ΔC13-CS-4S |
| ΔCS-4S | ΔC13-CS-4S |
| ΔCS-6S | ΔC13-CS-6S |
| ΔCS 2S4S | ΔC13-HS-2S6S |
| ΔCS-4S6S | ΔC13-CS-4S6S |
| ΔCS-2S6S | ΔC13-CS-4S6S |
| ΔCS-2S4S6S | ΔC13-HS-2SNS |
| ΔHA | ΔC13-HS-0S |

(C)

| Disaccharide | Q1 Mass (Da) | Q3 Mass (Da) | Entrance potential (V) | Declustering Potential (V) | Collision Energy (V) | Collision Cell Exit Potential (V) |
|---|---|---|---|---|---|---|
| **ΔCS-0S** | 572.1 | 396 | -10 | -33 | -35 | -31 |
| **ΔCS-2S** | 652.1 | 157 | -10 | -34 | -38 | -11 |
| **ΔCS-4S** | 652 | 536.2 | -10 | -33 | -42 | -34 |
| **ΔCS-6S** | 652.1 | 396 | -10 | -33 | -46 | -30 |
| **ΔCS 2S4S** | 732 | 652 | -10 | -27 | -25 | -26 |
| **ΔCS-4S6S** | 732 | 652 | -10 | -44 | -26 | -29 |
| **ΔCS-2S6S** | 732 | 652 | -10 | -43 | -22 | -30 |
| **ΔCS-2S4S6S** | 811.97 | 652 | -10 | -33 | -30 | -46 |
| **ΔHA** | 572.1 | 396 | -10 | -33 | -32 | -28 |
| **ΔC13-CS-0S** | 578.1 | 396 | -10 | -33 | -35 | -31 |
| **ΔC13-CS-4S** | 658 | 538.2 | -10 | -33 | -42 | -34 |
| **ΔC13-CS-6S** | 658.1 | 396 | -10 | -33 | -46 | -30 |
| **ΔC13-CS-4S6S** | 738 | 658 | -10 | -44 | -26 | -29 |
| **ΔC13-HS-0S** | 578.1 | 396 | -10 | -65 | -33 | -29 |
| **ΔC13-HS-2S6S** | 732 | 652 | -10 | -44 | -23 | -41 |
| **ΔC13-HS-2SNS** | 696 | 616 | -10 | -27 | -27 | -26 |

**Supplemental Table 4: Sampling probabilities used to calculate the inverse probability of selection weights in analyses of chondroitin sulfate and hyaluronic acid**

| Sampling Stratum | Included / Not Included | Baseline Analysis | 24-hour Analysis | 72-hour Analysis |
|---|---|---|---|---|
| Died within 90 days of randomization | Included | 275 | 220 | 147 |
| | Not Included | 66 | 121 | 194 |
| | Sampling Probability | 0.806 | 0.645 | 0.431 |
| Alive at 90 days with ARDS at baseline or within 7 days of baseline | Included | 29 | 28 | 27 |
| | Not Included | 4 | 5 | 6 |
| | Sampling Probability | 0.879 | 0.848 | 0.818 |
| Restrictive Arm - Alive at day 90 with no ARDS at randomization or by day 7 | Included | 135 | 112 | 70 |
| | Not Included | 454 | 469 | 413 |
| | Sampling Probability | 0.229 | 0.193 | 0.145 |
| Liberal Arm - Alive at day 90 with no ARDS at randomization or by day 7 | Included | 135 | 116 | 82 |
| | Not Included | 459 | 472 | 424 |
| | Sampling Probability | 0.227 | 0.197 | 0.162 |
| | Included Total | 574 | 476 | 326 |
| Not eligible: discharged prior to measurement time | | 0 | 14 | 194 |
| Not eligible: ARDS status at randomization or 7 days after randomization unknown | | 6 | 6 | 6 |

**Supplemental Table 5: Sampling probabilities used to calculate the inverse probability of selection weights in analyses of IL-6**

| Sampling Stratum | Included / Not included | Baseline Analysis | 72-hour Analysis |
|---|---|---|---|
| Died within 90 days of randomization | Included | 294 | 166 |
| | Not Included | 47 | 175 |
| | Sampling Probability | 0.862 | 0.487 |
| Alive at 90 days with ARDS at baseline or within 7 days of baseline | Included | 32 | 32 |
| | Not Included | 1 | 1 |
| | Sampling Probability | 0.97 | 0.97 |
| Restrictive Arm - Alive at day 90 with no ARDS at randomization or by day 7 | Included | 519 | 312 |
| | Not Included | 70 | 171 |
| | Sampling Probability | 0.881 | 0.646 |
| Liberal Arm - Alive at day 90 with no ARDS at randomization or by day 7 | Included | 524 | 334 |
| | Not Included | 70 | 172 |
| | Sampling Probability | 0.882 | 0.660 |
| | Included Total | 1369 | 844 |
| Not eligible: discharged prior to measurement time | | 0 | 194 |
| Not eligible: ARDS status at randomization or 7 days after randomization unknown | | 48 | 48 |

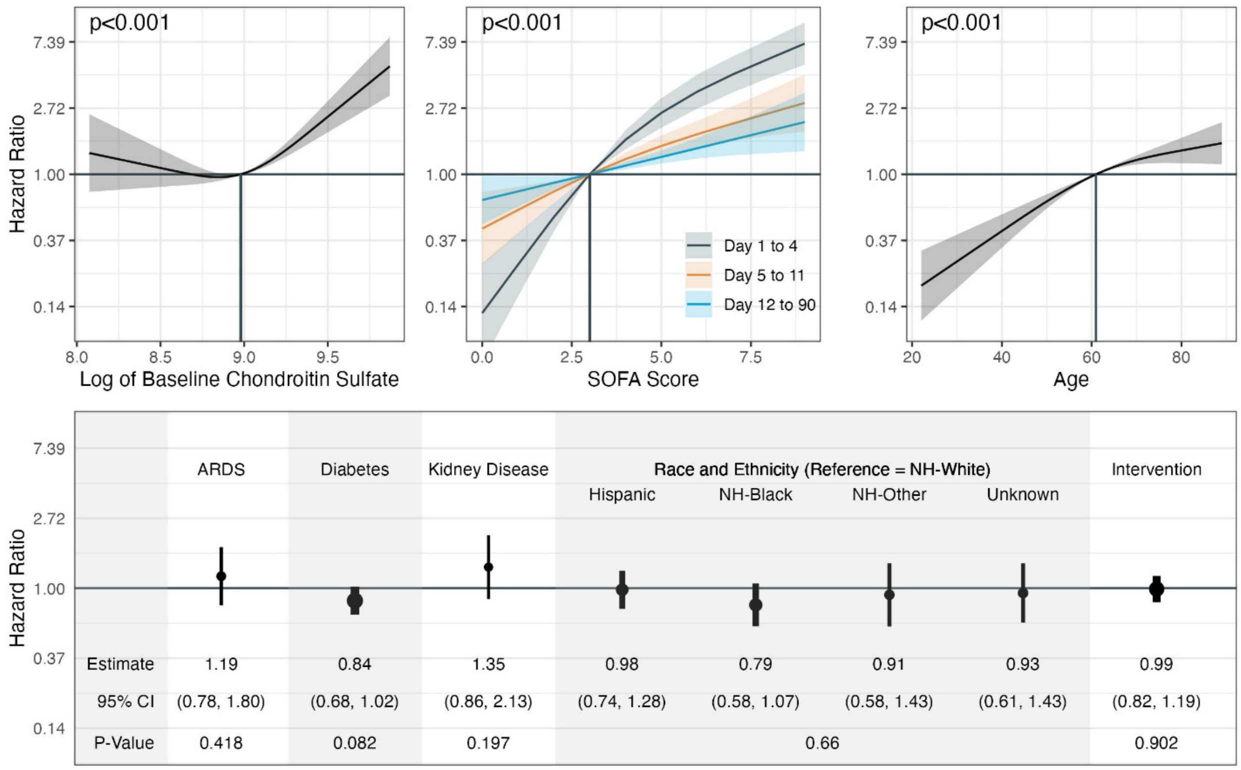


**Supplemental Figure 1. Baseline plasma chondroitin sulfate is an independent predictor of time to all-cause mortality within 90 days of CLOVERS randomization.** Partial effect plots for each variable for 90-day mortality in a Cox model stratified by sex assigned at birth and chronic heart failure using generalized raking. We chose a reference value for each variable and display the mean difference and pointwise 95% confidence intervals comparing all other values to the reference value. For quantitative variables, the reference value was the median value. The categorical variable panel displays hazard ratio estimates, 95% CIs, and p values, with confidence interval line thickness weighted proportionally to one divided by the estimated standard errors.

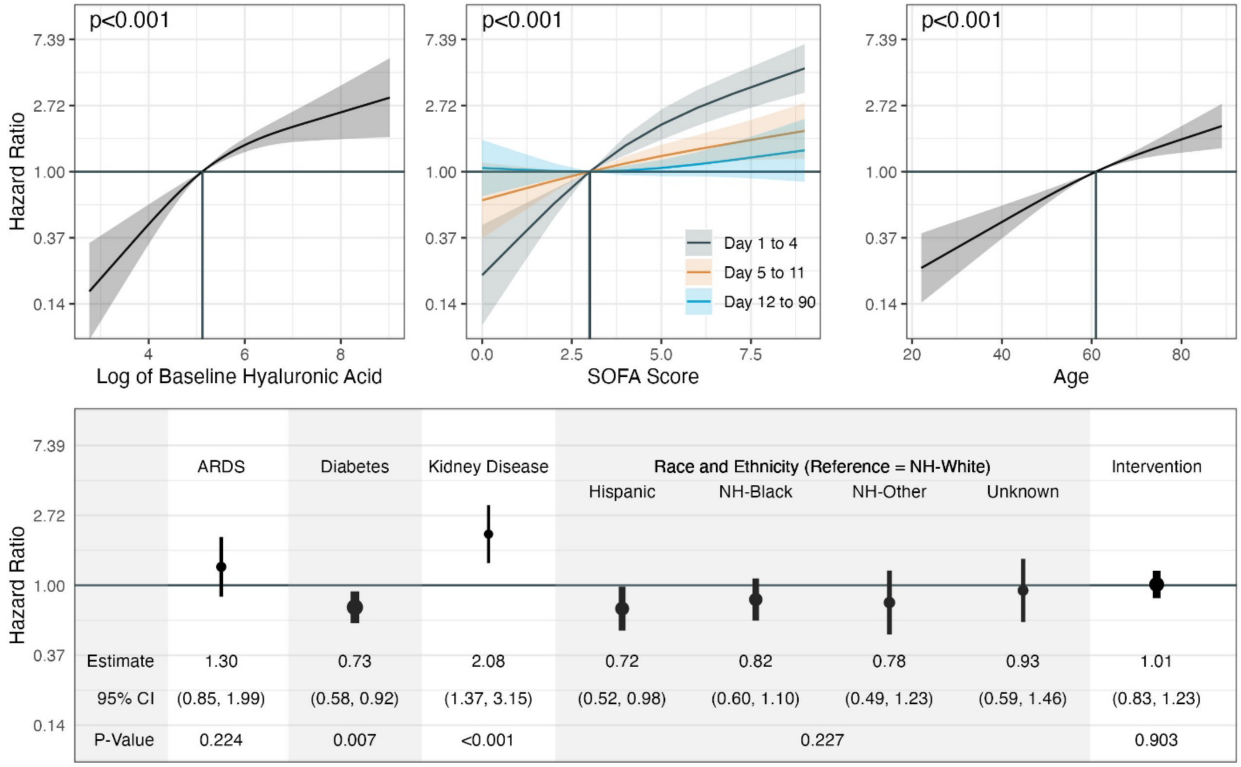


**Supplemental Figure 2. Baseline plasma hyaluronic acid is an independent predictor of time to all-cause mortality within 90 days of CLOVERS randomization.** Partial effect plots for each variable for 90-day mortality in a Cox model stratified by sex assigned at birth and chronic heart failure using generalized raking. We chose a reference value for each variable and display the mean difference and pointwise 95% confidence intervals comparing all other values to the reference value. For quantitative variables, the reference value was the median value. The categorical variable panel displays hazard ratio estimates, 95% CIs, and p values, with confidence interval line thickness weighted proportionally to one divided by the estimated standard errors.

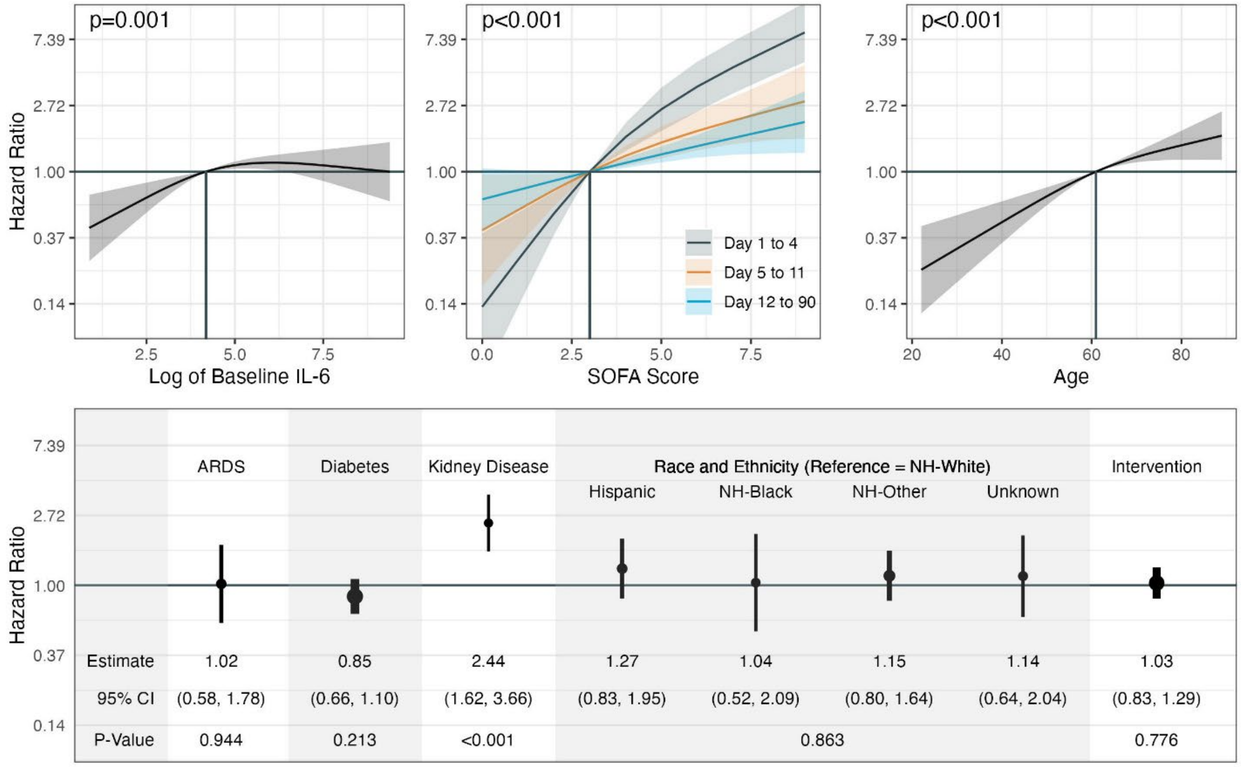


**Supplemental Figure 3. Baseline plasma IL-6 is an independent predictor of time to all-cause mortality within 90 days of CLOVERS randomization.** Partial effect plots for each variable for 90-day mortality in a Cox model stratified by sex assigned at birth and chronic heart failure using generalized raking. We chose a reference value for each variable and display the mean difference and pointwise 95% confidence intervals comparing all other values to the reference value. For quantitative variables, the reference value was the median value. The categorical variable panel displays hazard ratio estimates, 95% CIs, and p values, with confidence interval line thickness weighted proportionally to one divided by the estimated standard errors.

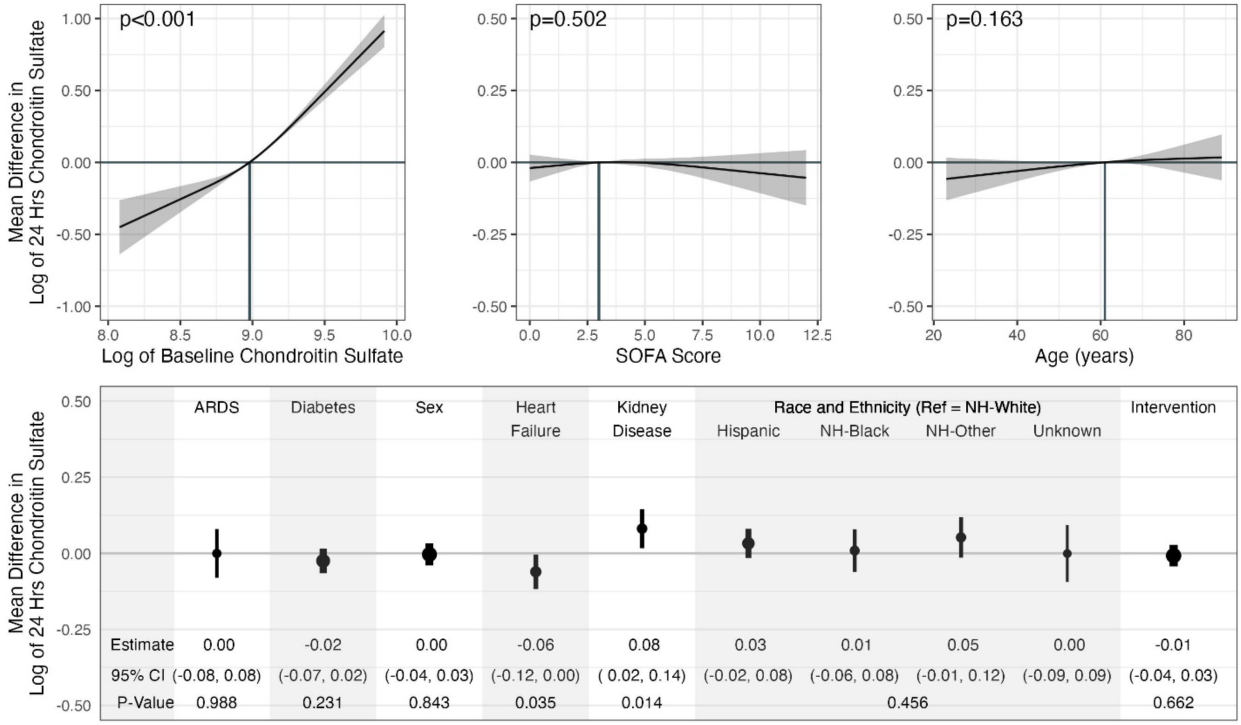


**Supplemental Figure 4. Randomization to a liberal or restrictive fluid-resuscitation approach has no impact on plasma chondroitin sulfate on multivariate analysis.** Inverse-probability weighted linear regression partial effect plots for the association between the change in chondroitin sulfate at 24 hours and baseline variables, including randomized treatment group and other baseline characteristics. We chose a reference value for each variable and display the mean difference and pointwise 95% confidence intervals comparing all other values to the reference value. For quantitative variables, the reference value was the median value. The categorical variable panel displays mean difference estimates, 95% CIs, and p values, with confidence interval line thickness weighted proportionally to one divided by the estimated standard errors.

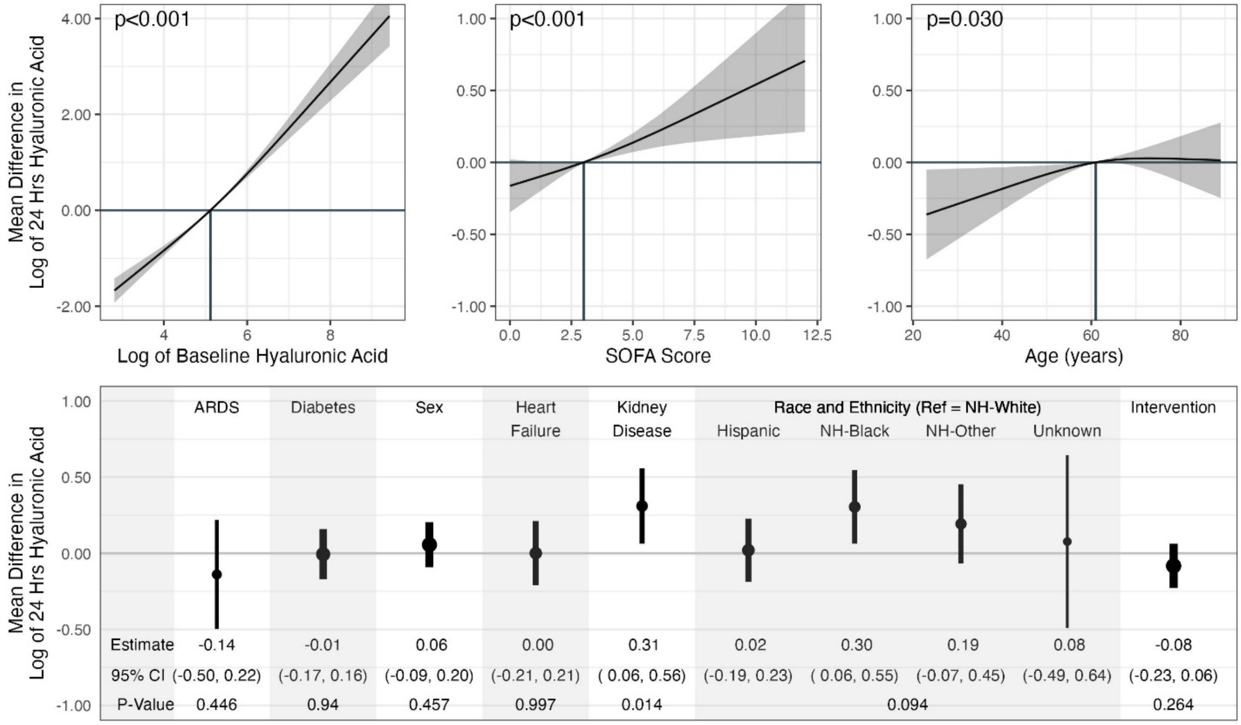


**Supplemental Figure 5. Randomization to a liberal or restrictive fluid-resuscitation approach has no impact on hyaluronic acid shedding on multivariate analysis.** Inverse-probability weighted linear regression partial effect plots for the association between the change in hyaluronic acid at 24 hours and baseline variables, including randomized treatment group and other baseline characteristics. We chose a reference value for each variable and display the mean difference and pointwise 95% confidence intervals comparing all other values to the reference value. For quantitative variables, the reference value was the median value. The categorical variable panel displays mean difference estimates, 95% CIs, and p values, with confidence interval line thickness weighted proportionally to one divided by the estimated standard errors.

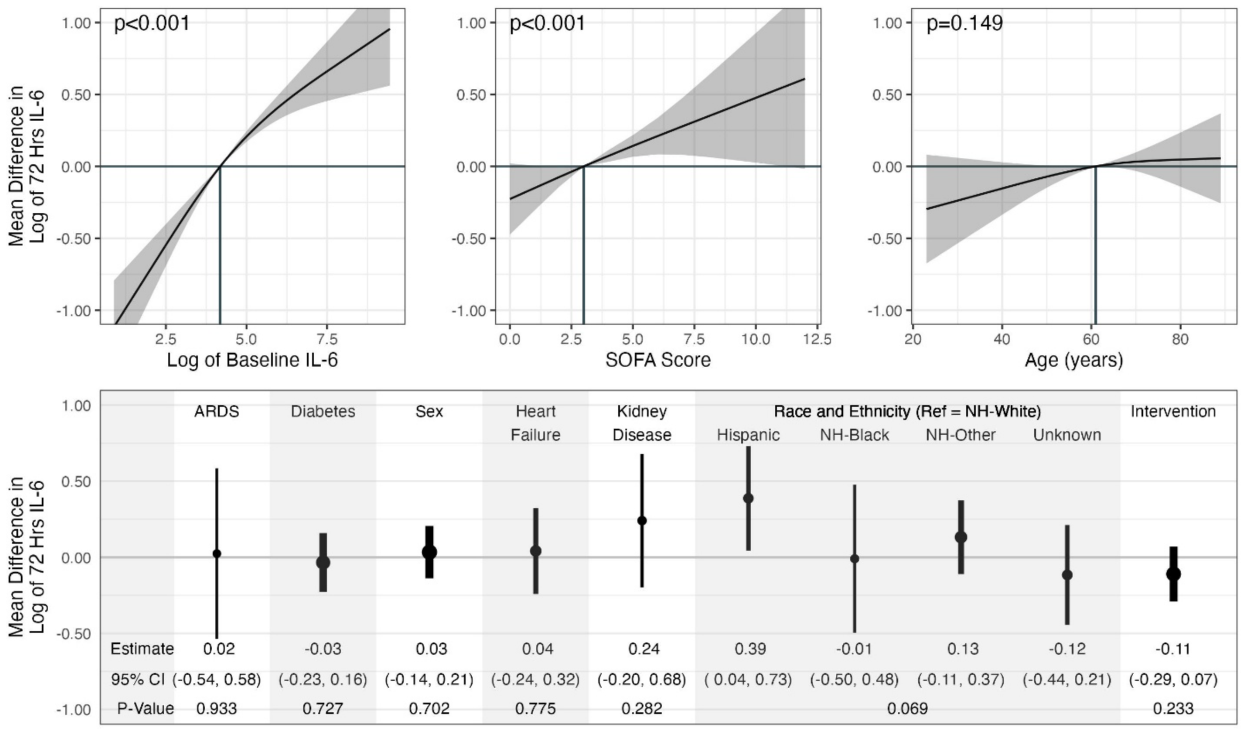


**Supplemental Figure 6. Randomization to a liberal or restrictive fluid-resuscitation approach has no impact on plasma IL-6 on multivariate analysis.** Inverse-probability weighted linear regression partial effect plots for the association between the change in IL-6 at 72 hours and baseline variables, including randomized treatment group and other baseline characteristics. We chose a reference value for each variable and display the mean difference and pointwise 95% confidence intervals comparing all other values to the reference value. For quantitative variables, the reference value was the median value. The categorical variable panel displays mean difference estimates, 95% CIs, and p values, with confidence interval line thickness weighted proportionally to one divided by the estimated standard errors.

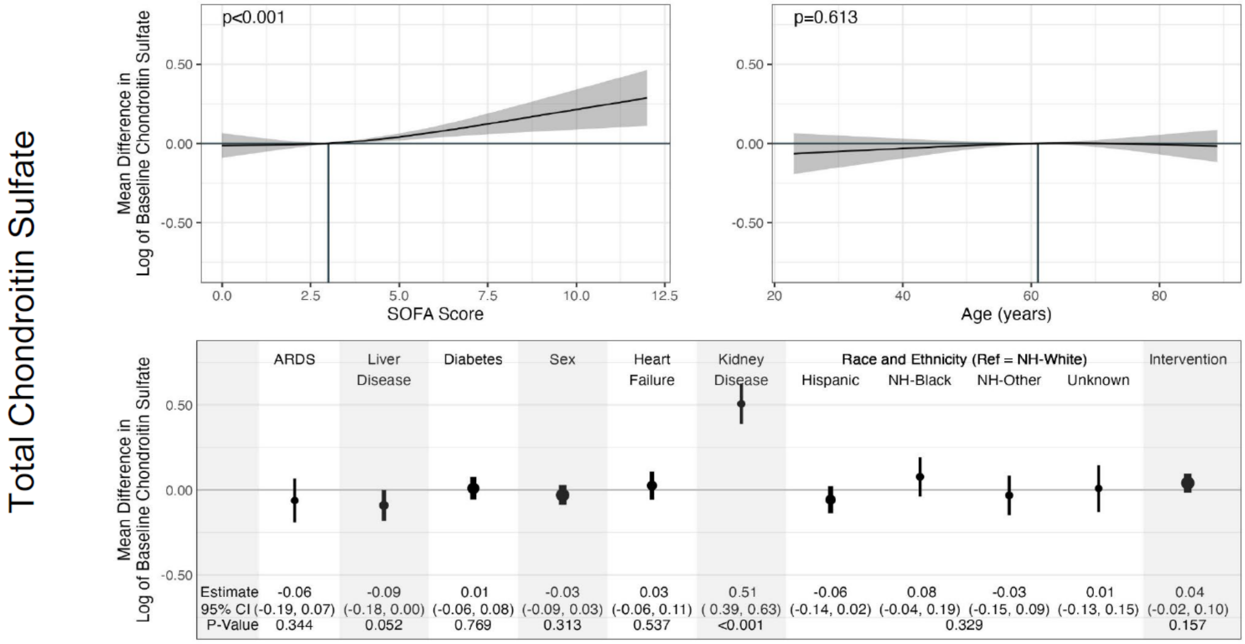


**Supplemental Figure 7. Association of baseline plasma concentrations of total chondroitin sulfate with patient characteristics.** Inverse-probability weighted linear regression partial effect plots for the association between baseline plasma total chondroitin sulfate concentrations and baseline variables, including randomized treatment group and other baseline characteristics. We chose a reference value for each variable and display the mean difference and pointwise 95% confidence intervals comparing all other values to the reference value. For quantitative variables, the reference value was the median value. The categorical variable panel displays mean difference estimates, 95% CIs, and p values, with confidence interval line thickness weighted proportionally to one divided by the estimated standard errors.

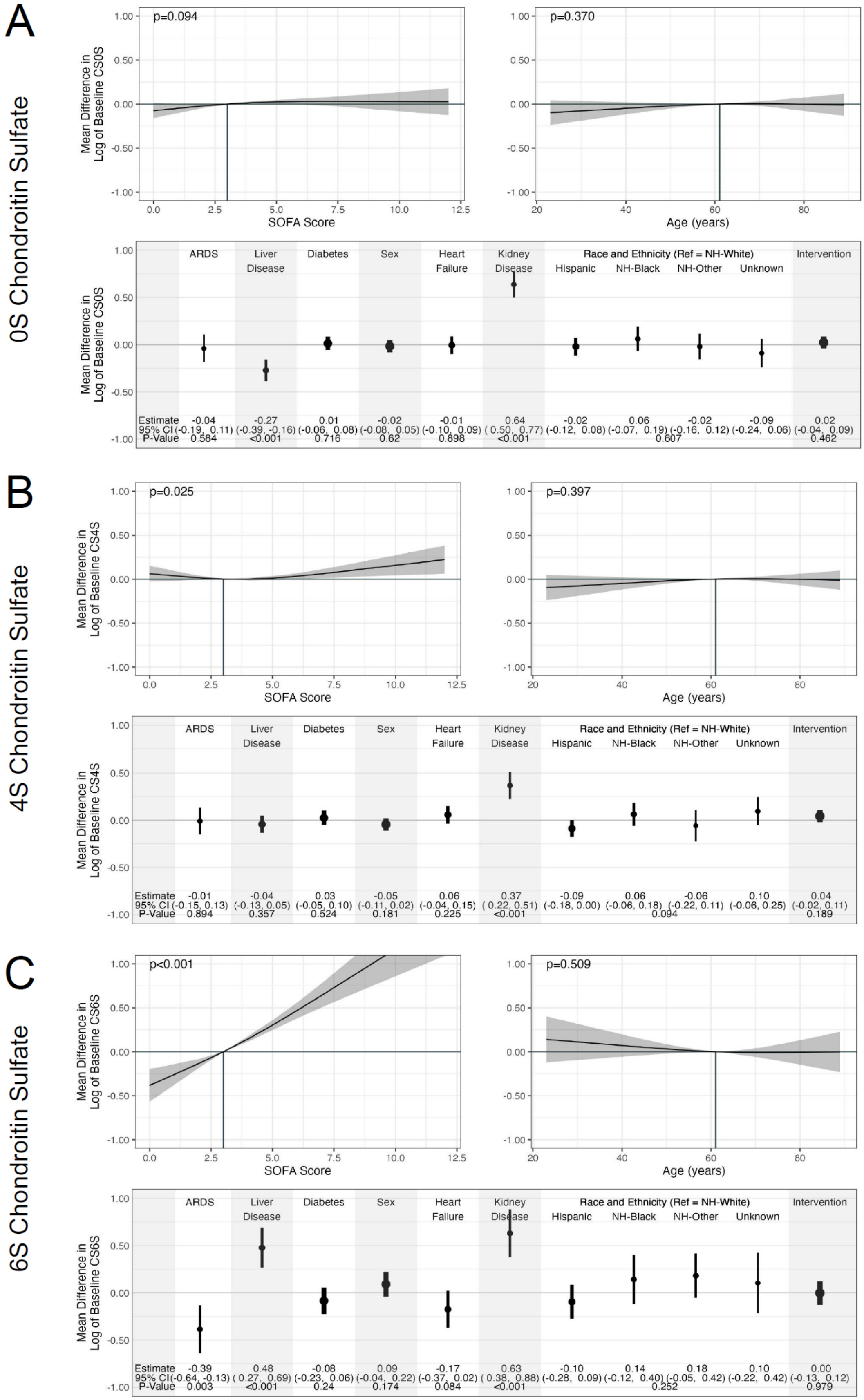
A
0S Chondroitin Sulfate
p=0.094
Mean Difference in Log of Baseline CS0S
SOFA Score
p=0.370
Age (years)
ARDS
Liver Disease
Diabetes
Sex
Heart Failure
Kidney Disease
Race and Ethnicity (Ref = NH-White)
Hispanic
NH-Black
NH-Other
Unknown
Intervention
Estimate -0.04 -0.27 0.01 -0.02 -0.01 0.64 -0.02 0.06 -0.02 -0.09 0.02
95% CI (-0.19, 0.11) (-0.39, -0.16) (-0.06, 0.08) (-0.08, 0.05) (-0.10, 0.09) ( 0.50, 0.77) (-0.12, 0.08) (-0.07, 0.19) (-0.16, 0.12) (-0.24, 0.06) (-0.04, 0.09)
P-Value 0.584 <0.001 0.716 0.62 0.898 <0.001 0.607 0.462
B
4S Chondroitin Sulfate
p=0.025
Mean Difference in Log of Baseline CS4S
SOFA Score
p=0.397
Age (years)
ARDS
Liver Disease
Diabetes
Sex
Heart Failure
Kidney Disease
Race and Ethnicity (Ref = NH-White)
Hispanic
NH-Black
NH-Other
Unknown
Intervention
Estimate -0.01 -0.04 0.03 -0.05 0.06 0.37 -0.09 0.06 -0.06 0.10 0.04
95% CI (-0.15, 0.13) (-0.13, 0.05) (-0.05, 0.10) (-0.11, 0.02) (-0.04, 0.15) ( 0.22, 0.51) (-0.18, 0.00) (-0.06, 0.18) (-0.22, 0.11) (-0.06, 0.25) (-0.02, 0.11)
P-Value 0.894 0.357 0.524 0.181 0.225 <0.001 0.094 0.189
C
6S Chondroitin Sulfate
p<0.001
Mean Difference in Log of Baseline CS6S
SOFA Score
p=0.509
Age (years)
ARDS
Liver Disease
Diabetes
Sex
Heart Failure
Kidney Disease
Race and Ethnicity (Ref = NH-White)
Hispanic
NH-Black
NH-Other
Unknown
Intervention
Estimate -0.39 0.48 -0.08 0.09 -0.17 0.63 -0.10 0.14 0.18 0.10 0.00
95% CI (-0.64, -0.13) ( 0.27, 0.69) (-0.23, 0.06) (-0.04, 0.22) (-0.37, 0.02) ( 0.38, 0.88) (-0.28, 0.09) (-0.12, 0.40) (-0.05, 0.42) (-0.22, 0.42) (-0.13, 0.12)
P-Value 0.003 <0.001 0.24 0.174 0.084 <0.001 0.252 0.979

**Supplemental Figure 8. Association of baseline plasma concentrations of chondroitin sulfate subtypes with patient characteristics.** Inverse-probability weighted linear regression partial effect plots for the association between baseline plasma (A) 0S chondroitin sulfate concentrations, (B) 4S chondroitin sulfate concentrations, (C) 6S chondroitin sulfate concentrations and baseline variables, including randomized treatment group and other baseline characteristics. We chose a reference value for each variable and display the mean difference and pointwise 95% confidence intervals comparing all other values to the reference value. For quantitative variables, the reference value was the median value. The categorical variable panel displays mean difference estimates, 95% CIs, and p values, with confidence interval line thickness weighted proportionally to one divided by the estimated standard errors.
